# Lower symptom burden strengthens the association between DTI-ALPS index and brain aging in head and neck cancer survivors

**DOI:** 10.1371/journal.pone.0360924

**Published:** 2026-11-13

**Authors:** Sahil Bajaj, Christopher Chad Quarles, Katherine Gilmore, Ying Qiao, Max Wintermark, Jeffrey S. Wefel, Kyle R. Noll, Chinmay Mokashi, Michelle Zalles, Michael Roth

**Affiliations:** 1 Department of Cancer Systems Imaging, Division of Diagnostic Imaging, The University of Texas MD Anderson Cancer Center, Houston, Texas, United States of America; 2 Cancer Neuroimaging Research Program, The University of Texas MD Anderson Cancer Center, Houston, Texas, United States of America; 3 Cancer Neuroscience Program, The University of Texas MD Anderson Cancer Center, Houston, Texas, United States of America; 4 Cancer Survivorship Program, The University of Texas MD Anderson Cancer Center, Houston, Texas, United States of America; 5 Department of Radiology, The University of Texas Medical Branch (UTMB), Galveston, Texas, United States of America; 6 Department of Neuro-Oncology, Division of Cancer Medicine, The University of Texas MD Anderson Cancer Center, Houston, Texas, United States of America; 7 Department of Pediatrics, Division of Pediatrics, The University of Texas MD Anderson Cancer Center, Houston, Texas, United States of America; Tianjin Medical University General Hospital, CHINA

## Abstract

**Background:**

Head and neck (HN) cancer survivorship is associated with persistent symptom burden and long-term brain-behavioral health alterations, yet mechanisms underlying brain aging remain poorly understood. We investigated whether HN cancer survivors exhibit accelerated brain aging, whether limbic white matter (WM) pathways contribute to these changes, and whether measures associated with glymphatic function are associated with reduced brain aging.

**Methods:**

Diffusion MRI and symptom burden data were analyzed in 135 HN cancer survivors and compared with a normative cohort from the Human Connectome Project (n = 1,228). Two machine learning models (elastic net [ENET] and random forest [RF]) using fractional anisotropy (FA) and quantitative anisotropy (QA) from 45 WM tracts were trained to estimate brain age and compute brain age gap (BAG). Glymphatic-related processes were indirectly assessed using diffusion tensor imaging analysis along the perivascular spaces (DTI-ALPS). Moderation analyses examined whether symptom burden influenced associations among DTI-ALPS, BAG, and WM integrity.

**Results:**

Survivors showed a negative mean BAG of −1.64 years in the ENET model, indicating a nonsignificant tendency toward delayed brain aging, whereas the RF model showed a positive mean BAG of 3.59 years, indicating accelerated brain aging. The RF model demonstrated stronger external generalizability and greater biological coherence, including associations with ALPS and symptom-related measures. The bilateral fornix and right U-fibers were among the strongest contributors to RF-based brain age prediction. Higher DTI-ALPS index values were associated with lower BAG and greater fornix integrity, particularly in survivors with lower symptom burden and reduced activity-related interference.

**Conclusions:**

HN cancer survivorship was associated with accelerated brain aging in the model demonstrating greater external generalizability and biological relevance, alongside alterations in limbic WM pathways. Higher DTI-ALPS was associated with reduced brain aging, with symptom burden modifying this relationship. These findings highlight brain health as an important component of cancer survivorship.

## Introduction

Head and neck (HN) cancer survivors experience persistent behavioral and affective symptoms (including fatigue, sleep disturbance, cognitive inefficiency, and emotional dysregulation) that may persist for years after treatment completion [1–3]. These symptoms adversely affect quality of life, functional independence, and return to work, yet the neurobiological mechanisms underlying their persistence remain incompletely understood. Emerging evidence suggests that such chronic symptom burden may reflect cumulative alterations in brain health, including processes related to accelerated brain aging [4,5].

Accumulating data indicate that cancer and its treatments exert widespread effects on the central nervous system [6,7]. Chemotherapy-related neurotoxicity, radiation-induced vascular and inflammatory changes, chronic psychosocial stress, sleep disruption, and immune dysregulation have all been implicated in altered brain structure and function among cancer survivors [8–12]. However, neuroimaging studies in HN cancer have largely focused on focal cortical abnormalities or gross volumetric changes, providing limited insight into distributed white matter (WM) alterations that may underlie persistent symptom burden and age-related neural vulnerability. In other contexts, including aging and neurodegenerative conditions, alterations in diffusion indices – such as fractional anisotropy [FA] and quantitative anisotropy [QA]) – have been linked to accelerated biological brain aging and functional impairment [13,14]. Yet, whether HN survivorship is associated with measurable deviations from normative brain aging trajectories remains unknown.

In cancer survivorship, distributed brain disruptions may reflect the combined effects of treatment-related toxicity, neuroinflammation, vascular compromise, and altered metabolic homeostasis—processes that may converge to accelerate brain aging [4,5,15]. To quantify such deviations, machine learning–based brain age modeling has emerged as a powerful framework for estimating biological brain age from neuroimaging data [16,17]. By training predictive models on diffusion MRI features from healthy adults, biological brain age can be estimated and compared with chronological age to derive the brain age gap (BAG; predicted age minus chronological age) [18]. A positive BAG reflects an older-appearing brain relative to normative expectations and has been associated with cognitive decline and neuropsychiatric health [18,19]. Diffusion-based brain age models are particularly sensitive to distributed WM microstructural alterations [17,20], making them well suited to detect measurable, network-level neural aging effects in cancer survivorship.

A biologically meaningful brain age framework requires anchoring predictions to a robust normative reference. Large-scale healthy aging datasets, such as the Human Connectome Project–Aging (HCP-A) [21,22] cohort, provide high-quality diffusion MRI data spanning midlife through older adulthood. By learning multivariate relationships between WM microstructure and chronological age in a neurologically healthy population, normative models establish expected trajectories of WM aging. These trajectories allow quantification of disease- or treatment-related deviations in clinical cohorts [23,24].

WM aging and vulnerability are not uniform across the brain. Limbic and long-range association pathways (including the fornix, cingulum, corticostriatal, corticothalamic, and short-range U-fiber systems) are particularly susceptible to microstructural degradation and support memory, executive function, affect regulation, and sensorimotor integration [25–29]. Disruption of these pathways has been linked to fatigue, cognitive inefficiency, and affective symptoms across aging and clinical populations [26,30–32]. In HN cancer survivorship, microstructural compromise within these networks may therefore contribute to accelerated brain aging and persistent symptom burden. In parallel, growing attention has focused on the glymphatic system—a brain-wide perivascular clearance network that facilitates cerebrospinal fluid–mediated removal of metabolic waste and inflammatory byproducts [33–35]. Glymphatic-related processes can be indirectly assessed using the diffusion MRI–based analysis along the perivascular space (DTI-ALPS) index [36]. The DTI-ALPS index is strongly influenced by sleep quality, vascular integrity, and inflammatory status [37]—domains frequently disrupted in cancer survivors. Impaired glymphatic-related processes have been linked to WM degeneration and accelerated brain aging [38–40], whereas preserved glymphatic-related processes may support neural resilience by maintaining metabolic homeostasis and limiting inflammatory burden [41–43]. Critically, the neuroprotective influence of such processes may not operate uniformly across individuals but instead depend on clinical context. Symptom burden—particularly sleep disturbance, fatigue, and affective interference—may reflect physiological states that compromise glymphatic-related processes or overwhelm its protective effects. Thus, symptom burden may moderate the relationship between DTI-ALPS index and brain aging, such that greater index values provide stronger associations at lower levels of symptom burden, whereas this association weakens as symptom burden increases. Critically, this moderation hypothesis has not yet been empirically tested in the context of ALPS index and diffusion-based BAG across distributed WM pathways in HN cancer survivorship.

We *hypothesized* that (a) HN cancer survivors would exhibit accelerated brain aging relative to normative expectations; (b) limbic and association pathways would contribute disproportionately to brain age estimation; (c) symptom burden would significantly moderate the relationship between ALPS index and BAG, such that stronger associations between ALPS index and accelerated brain aging would be observed among survivors with lower symptom burden; and (d) higher ALPS index would be positively associated with greater microstructural integrity (FA and QA) within limbic and association WM pathways, with these positive associations being strongest among survivors with lower symptom burden.

## Materials and methods

### Data collection

***Participants.*** The study cohort comprised 1,228 healthy-controls (HCs) drawn from a large, demographically diverse, community-dwelling cohort suitable for lifespan modeling, the Aging Adult Brain Connectome (AABC) Release 1 via the Lifespan HCP-Aging cohort [21,22] (age range = 36 to ≥90 years; mean age = 61.75 years, SD = 16.00; 525 males) and 135 long-term survivors of HN cancer (age range = 22.18–93.56 years; mean age = 66.04 years, SD = 11.83; 107 males) evaluated during the survivorship phase of care.

HCs from the HCP-Aging cohort had no history of neurological, psychiatric, or major medical disorders and no contraindications to MRI [21]. All HN participants had completed primary cancer treatment and showed no clinical evidence of recurrent or metastatic disease at the time of imaging. HN participants represented a wide range of survivorship duration, with MRI acquired on average 7.14 years following initial cancer diagnosis. Diagnosis dates were unavailable for a small subset of participants (n = 6). Exclusion criteria included a history of neurological or psychiatric disorders unrelated to cancer, prior traumatic brain injury, or contraindications to MRI. See Table 1 for participant characteristics in detail and Supplementary section S1 File for tumor and treatment characteristics of HN participants.

**Table 1:** Participant characteristics.

Characteristics			HCs (HCP Cohort)	HN Cohort	Statistics (Two sample t-test/Chi-squared
**Before Age Harmonization (N = 1,228 for HCs & 135 for HN survivors)**					
Mean Age in years (SD) at		MRI scan	61.75 (16.00)	66.04 (11.83)	t (192.4) = -3.85, *p* < .0001
		MDASI Data Collection	N/A	67.87 (10.94)	N/A
		Diagnosis*	N/A	58.90 (11.59)	N/A
Sex (M/F)			525/703	107/28	c^2^(1) = 64.5, *p <* .0001
Average Time** (SD) in years between MRI Scan and MDASI Data Collection			N/A	1.80 (4.82)	N/A
**Symptom Severity**					
Mean Core Symptom Severity (SD)			N/A	1.57 (1.49)	N/A
Mean HN-specific Symptom Severity (SD)			N/A	1.52 (1.60)	N/A
**Symptom Interference**					
Mean Activity-related Symptom Inference (SD)			N/A	1.41 (2.17)	N/A
Mean Affective Symptom Inference (SD)			N/A	1.07 (1.88)	N/A
**Following Age Harmonization (N = 1,176 for HCs & 132 for HN survivors)**					
Mean Age in years (SD) at	MRI scan		60.51 (15.20)	66.43 (10.65)	t (196.6) = -5.77, *p* < .0001
	MDASI Data Collection		N/A	68.22 (9.99)	N/A
	Diagnosis*		N/A	65.09 (10.82)	N/A
Sex (M/F)			502/674	105/27	χ²(1) = 64.7, p < .0001
Average Time** (SD) in years between MRI Scan and MDASI Data Collection			N/A	1.76 (4.83)	N/A
**Symptom Severity**					
Mean Core Symptom Severity (SD)			N/A	1.57 (1.50)	N/A
Mean HN-specific Symptom Severity (SD)			N/A	1.54 (1.61)	N/A
**Symptom Interference**					
Mean Activity-related Symptom Inference (SD)			N/A	1.39 (2.14)	N/A
Mean Affective Symptom Inference (SD)			N/A	1.02 (1.73)	N/A

*Diagnosis date was not available for 6 patients.

**Positive values indicate that the MRI scan was acquired before MDASI data collection, whereas negative values indicate that the MRI scan occurred after MDASI data collection.

All data were obtained retrospectively from institutional medical records under protocols approved by the Institutional Review Board at The University of Texas MD Anderson Cancer Center, with informed consent waived. Data were accessed for research purposes on December 21, 2024. The authors had access to information that could identify individual participants during and/or after data collection.

***Symptom Burden Assessment.*** Multidimensional symptom burden was assessed using the MD Anderson Symptom Inventory (MDASI) [44] and the MDASI Head and Neck Module (MDASI-HN) [45]. Symptom burden encompassed both severity and interference, reflecting symptom intensity and their impact on daily functioning. The core MDASI includes 13 common cancer-related symptoms and 6 interference items, capturing activity-related (e.g., general activity, work, walking) and affective (e.g., mood, enjoyment of life, relationships) domains. The MDASI-HN extends the core instrument with 9 head and neck–specific symptoms, enabling comprehensive assessment in this population. All items are rated on an 11-point scale (0–10) based on the past 24 hours. Subscale scores were calculated as the mean of items within each domain, yielding summary measures of core symptom severity, HN-specific symptom severity, activity-related interference, and affective interference, with higher scores indicating greater symptom burden. Both the MDASI and MDASI-HN demonstrate strong internal consistency reliability, with Cronbach’s α values ranging from 0.83 to 0.94 across domains [44,45].

***MRI Acquisition.*** Diffusion MRI data from HN cancer survivors were collected retrospectively as part of routine clinical imaging on a 3 Tesla GE Signa scanner equipped with a 24-channel head coil. Imaging employed single-shot echo-planar diffusion-weighted sequences optimized for clinical feasibility. Typical acquisition parameters included a repetition time (TR) of ~8,000 ms, echo time (TE) of ~70 ms, flip angle of 90°, voxel dimensions of approximately 0.9 × 0.9 × 5.0 mm^3^, and either 20 or 27 diffusion-weighted directions (b = 1,000 or 1,200 s/mm^2^), along with 1–3 non-diffusion-weighted (b0) volumes. Diffusion MRI data from HCP cohort were acquired using the standardized HCP-Aging imaging protocol (see Harms et. al. for details [22]) and served as the normative reference sample for model training.

### Data analysis

#### Diffusion MRI reconstruction and tractography.

***Image Preprocessing.*** Diffusion MRI data were corrected for head motion using FSL [46]. Further processing was performed using DSI Studio [47,48], which began with the generation of a source (SRC) reconstruction file from the NIFTI data. A rigorous quality-control (QC) procedure was implemented to ensure the integrity and quality of the diffusion data and SRC files. QC included verification of image dimensions, the number of diffusion-weighted images (DWIs), and the number of diffusion shells, as inconsistencies in these parameters could indicate incomplete or corrupted acquisitions. In addition, DSI Studio was used to identify low-quality outlier DWIs based on neighboring DWI correlation, a metric sensitive to artifacts associated with eddy currents, head motion, and potential head-coil malfunctions. Datasets with a neighboring DWI correlation <0.7 underwent manual visual inspection to determine whether the observed artifacts were correctable. Datasets with uncorrectable artifacts or otherwise inadequate image quality were excluded from subsequent analyses. All datasets included in the present study met the predefined QC criteria and were retained for subsequent processing and analysis.

***WM Pathways and Feature Extraction.*** Microstructural features of WM were quantified using diffusion tensor imaging, including FA and QA. Unlike FA, QA is derived from Q-space diffeomorphic reconstruction [49], which better models complex fiber orientations (e.g., crossing or bending tracts) and reduces partial volume effects, resulting in enhanced spatial resolution and more reliable tract delineation. A diffusion sampling length ratio of 1.25 was applied, with an output resolution of 2-mm isotropic voxels. Unless otherwise specified, tractography was performed using the default parameters implemented in DSI Studio. These included automated determination of the number of seeds and tracts based on the seed-to-voxel ratio; Otsu-based anisotropy thresholding with randomized selection of 0.5–0.7 of the resulting threshold; randomized angular thresholds ranging from 45° to 90°; a step size corresponding to the voxel spacing; no smoothing; minimum and maximum tract lengths of 30 and 200 mm, respectively; and a default random seed of zero. Mean FA and QA values were computed across 45 major WM fiber bundles (see Table 2 for the detailed list) defined by the HCP842 atlas [50], providing tract-wise measures of WM structure and integrity. These diffusion-derived metrics were used as inputs for brain age prediction modeling and pathway-specific analyses investigating contributors to accelerated aging and DTI-ALPS index.

**Table 2:** White-matter (WM) fiber bundles of interest.

21 Bilateral and 3 Midline Major WM Bundles of Interest (Total = 45)
**Projection Pathways (Bilateral)** Acoustic Radiation Corticospinal Tract Corticostriatal Pathway Corticothalamic Pathway Fornix Optic Radiation Frontopontine Tract Occipitopontine Tract Parietopontine Tract Temporopontine Tract **Association Pathways (Bilateral)** Arcuate Fasciculus Cingulum Extreme Capsule Frontal Aslant Tract Inferior Fronto Occipital Fasciculus Inferior Longitudinal Fasciculus Middle Longitudinal Fasciculus Superior Longitudinal Fasciculus U-fiber Uncinate Fasciculus Vertical Occipital Fasciculus **Commissural Pathways (Midline)** Anterior Commissure Corpus Callosum Posterior Commissure

***Glymphatic Function Assessment.*** Glymphatic function was indirectly assessed using the DTI-ALPS index [36,51]. The DTI-ALPS index is an indirect diffusion MRI metric derived from directional diffusivities in specific periventricular white matter regions and has been proposed as a marker of glymphatic-related processes. Importantly, the ALPS index may also be influenced by local white matter fiber architecture, brain atrophy, ventricular enlargement, white matter lesions, region of interest (ROI) placement, partial-volume effects, and diffusion acquisition parameters [52–54]. Diffusivity measures along orthogonal axes were extracted from regions adjacent to the lateral ventricles, corresponding to projection and association fiber areas oriented relative to the perivascular spaces. The ALPS index was calculated as the ratio of diffusivity along the perivascular direction to diffusivity orthogonal to these spaces, with higher values generally interpreted as reflecting greater glymphatic-related transport. All ROI placements and ALPS calculations were visually inspected to ensure anatomical accuracy and data quality.

***Brain Age Prediction Framework.*** Diffusion MRI data from a healthy control (HC) cohort (HCP-Aging) [21] and an external evaluation/validation HN cancer cohort (MD Anderson) were used to develop and validate brain age models. Analyses were restricted to a single time point, with biological sex included as a predictor. Age ranges were harmonized using age-range restriction approach across cohorts to ensure valid generalization. HN cancer participants whose ages fell outside the minimum-to-maximum age range of the HC cohort were excluded, reducing the HN cohort from 135 to 132 participants. The HC cohort was then restricted to participants within the resulting age range of the HN cohort, reducing the cohort from 1,228–1,176 participants. This procedure ensured that both cohorts were analyzed within a common overlapping age range and minimized extrapolation of the brain age model beyond the represented age range. The HC cohort was used for model development, and the HN cohort served as an external validation dataset. FA and QA were extracted from 45 WM bundles (90 features total). After removing missing and zero-variance features, continuous variables were z-scored within cross-validation folds to prevent data leakage. The HC cohort was split into training (80%) and held-out test (20%) sets using age-stratified sampling. Model development employed nested age-stratified cross-validation (10 × 10), with out-of-fold predictions used for unbiased performance estimation and bias correction. Elastic Net (ENET) regression [55] and Random Forest (RF) regression [56] models were trained with hyperparameters optimized via grid search. Final models were retrained on the full training set and evaluated on both the held-out HC test set and the external validation HN cohort. Regression dilution bias was corrected using a linear model derived from training predictions, and BAG was computed as the difference between predicted and chronological age. Performance was assessed using Pearson correlation (r), coefficient of determination (R^2^), mean absolute error (MAE), and root mean squared error (RMSE). Feature importance was evaluated using model-specific metrics, with key features identified via a knee-point approach. Scanner harmonization between HC and HN cohorts was not included in the primary analysis, as sensitivity analyses indicated reduced predictive performance following harmonization. Also, in adult cohorts, the brain–age signal is relatively weak and less confounded with site, so harmonization methods may remove limited noise while attenuating true biological variability, resulting in minimal or no improvement in prediction. This framework emphasizes strict data separation, nested validation, and bias correction to ensure robust generalization.

***Symptom Burden as a Moderator of ALPS–BAG and Microstructural Associations.*** Moderation analyses were conducted to test whether symptom burden influenced the association between ALPS index and BAG, using PROCESS (v4.2) in SPSS (v29). Separate models were run for each symptom domain (core severity, HN-specific severity, activity-related interference, and affective interference). All models adjusted for chronological age, sex, and the interval between MRI and symptom assessment. Continuous variables were mean centered prior to interaction testing. The omnibus regression models and moderation was considered significant at Bonferroni-corrected threshold of *p* < .0125 (α = .05/4) to account for multiple comparisons across the four symptom burden domains rather than across ML models, because ENET and RF were evaluated as complementary, parallel modeling approaches rather than as independent hypothesis tests of the same primary effect. Significant effects were probed using simple slopes and interaction plots. To further examine potential underlying mechanisms, feature-level moderation analyses were performed to assess whether symptom burden moderated the associations between the ALPS index and WM microstructure. Analyses were restricted to the models showing significant ALPS × symptom burden interactions at *p* < .0125 in the primary ALPS–BAG moderation analyses. The WM pathways identified as key contributors to these brain age models using the knee-point feature selection method were then examined, with correction for multiple comparisons applied across the feature-level analyses.

## Results

### Participant characteristics and covariate specification

Following age harmonization, healthy controls (HCs; *n* = 1,176) and HN survivors (*n* = 132) differed significantly in age and sex. HCs were younger (36–88 years) than HN survivors (37.40–88.39 years; *t*(196.6) = –5.77, *p* < .0001), and HN survivors had a higher proportion of males (79.5% vs. 42.7%; χ^2^(1) = 64.7, *p* < .0001). Accordingly, age and sex were included as covariates in all analyses. The mean interval between MRI and MDASI assessment was 1.76 years. Given that MDASI captures persistent treatment-related symptoms despite assessing the prior 24 hours [57], this interval was included as a covariate in analyses involving both imaging and symptom measures. See Table 1 for detailed participant characteristics.

### Brain age predictions and BAG

Bias-corrected performance metrics demonstrated strong brain age prediction performance in HCs and reduced generalization in the external HN cohort. For the ENET model, performance in the HC training set (N = 941) showed a high correlation between predicted and chronological age (R = 0.88, R^2^ = 0.77) with low error (MAE = 5.69 years, RMSE = 7.22 years) and a near-zero mean BAG (0.06 ± 7.23 years). Similar performance was observed in the HC test set (N = 235), with R = 0.87, R^2^ = 0.76, MAE = 5.80 years, RMSE = 7.41 years, and a slightly positive BAG (0.23 ± 7.43 years). However, in the external HN validation cohort (N = 132), model performance was R = 0.60 and R^2^ = −0.66, with MAE = 10.93 years, RMSE = 13.66 years, and a negative mean BAG of −1.64 ± 13.61 years, indicating delayed brain aging in the HN cohort relative to HCs. For the RF model, performance in the HC training set (N = 941) was slightly lower than ENET but still robust (R = 0.81, R^2^ = 0.65, MAE = 6.96 years, RMSE = 8.91 years, BAG = 0.10 ± 8.92 years). Comparable results were observed in the HC test set (N = 235) with R = 0.80, R^2^ = 0.64, MAE = 7.32 years, RMSE = 9.12 years, and a modestly positive BAG (0.54 ± 9.13 years). Notably, in the external validation HN cohort (N = 132), RF showed improved generalization relative to ENET, with R = 0.60 and R^2^ = 0.24, with lower prediction error (MAE = 7.35 years, RMSE = 9.25 years), and a positive mean BAG of 3.59 ± 8.56 years, suggesting accelerated brain aging in the HN cohort relative to HCs.

Accordingly, both candidate models were retained for subsequent clinical and biological analyses based on complementary evidence of predictive performance across the evaluation cohorts. The ENET model demonstrated stronger internal predictive performance in the held-out HC sample, whereas the RF model demonstrated better external generalizability in the subsequent NF1 clinical cohort. In the HN cancer cohort, the ENET model yielded a negative mean BAG (95% CI: −3.99 to 0.70), which was not significantly different from zero (one-sample *t*-test: *t*_131_ = −1.39, *p* = .17). In contrast, the RF model yielded a positive mean BAG (95% CI: 2.11–5.06), which was significantly greater than zero (one-sample *t*-test: *t*_131_ = 4.82, *p* < .001). Rather than selecting a single model based on performance in either held-out HC sample or external clinical dataset, both models were carried forward to determine whether their derived brain-age measures demonstrated consistent or model-specific relationships with clinical and biological measures. Importantly, neither model was retrained or refitted using clinical patient data; model parameters were derived entirely from the HC cohort and subsequently applied to the clinical cohorts. This approach allowed the downstream analyses to evaluate the robustness and model-specificity of brain-age associations across the two modeling approaches.

Fig 1A–1C summarize the ENET brain age model, showing the association between predicted and chronological age (Fig 1A), the distribution of bias-corrected BAG values (Fig 1B), and the top 10 WM pathway contributors with their feature-importance scores (Fig 1C). The model identified a distributed set of WM pathways as contributors to brain age prediction. Using the knee-point selection method, the feature set was reduced to the top three predictors: bilateral corticothalamic pathways (QA) and the right corticothalamic pathway (FA). Fig 2A–2C present the corresponding results for the RF brain age model, including predicted versus chronological age (Fig 2A), the distribution of bias-corrected BAG values (Fig 2B), and the top 10 WM pathway contributors with their feature-importance scores (Fig 2C). The knee-point method likewise identified three key predictors: bilateral fornix (QA) and right U-fibers (FA). The anatomical locations of top three contributors are shown in Fig 3 (ENET model) and Fig 4 (RF model).

**Fig 1:**
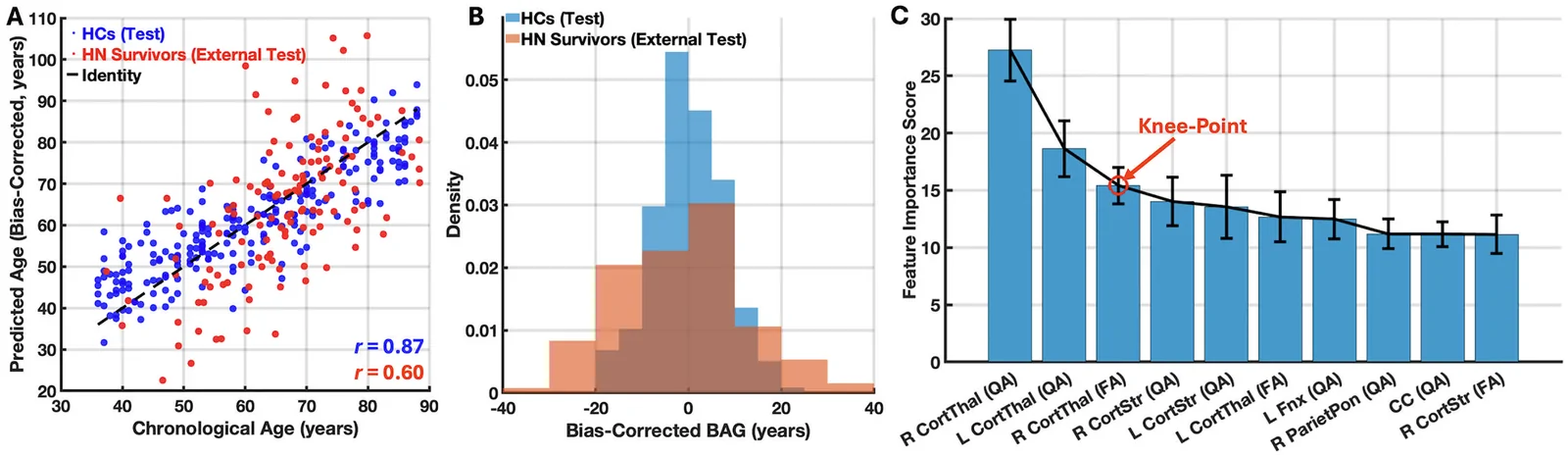
Elastic Net (ENET) brain age model results. (A) Association between ENET-predicted brain age and chronological age. (B) Distribution of bias-corrected BAG values. (C) Top WM pathway contributors ranked by feature importance scores. (Abbreviations: QA: Quantitative Anisotropy; FA: Fractional Anisotropy; R CortThal: Right Corticothalamic Pathways; L CortThal: Left Corticothalamic Pathways; R CortStr: Right Corticostriatal Pathways; L CortStr: Left Corticostriatal Pathways; L Fnx: Left Fornix; R ParietPon: Right Parietopontine Tracts; CC: Corpus Callosum).

**Fig 2:**
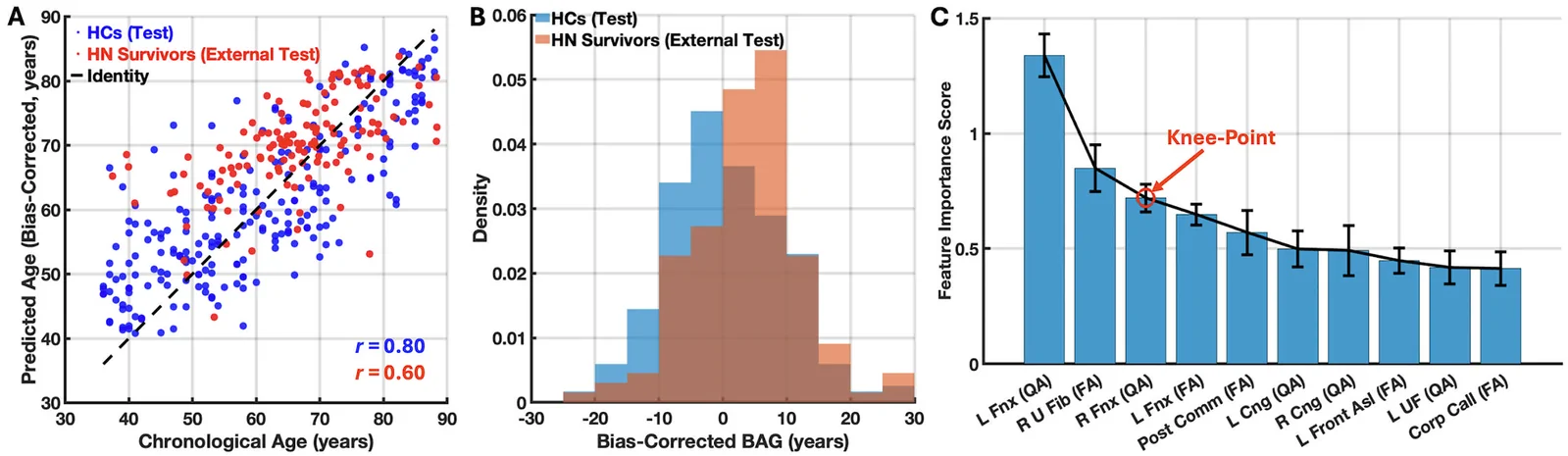
Random forest (RF) brain age model results. (A) Association between RF-predicted brain age and chronological age. (B) Distribution of bias-corrected BAG values. (C) Top WM pathway contributors ranked by feature importance scores. (Abbreviations: QA: Quantitative Anisotropy; FA: Fractional Anisotropy; L Fnx: Left Fornix; R U Fib: Right U-Shaped Fibers; R Fnx: Right Fornix; Post Comm: Posterior Commissure; L Cng: Left Cingulum; R Cng: Right Cingulum; L Front Asl: Left Frontal Aslant Tract; L UF: Left Uncinate Fasciculus; and CC: Corpus Callosum).

**Fig 3:**
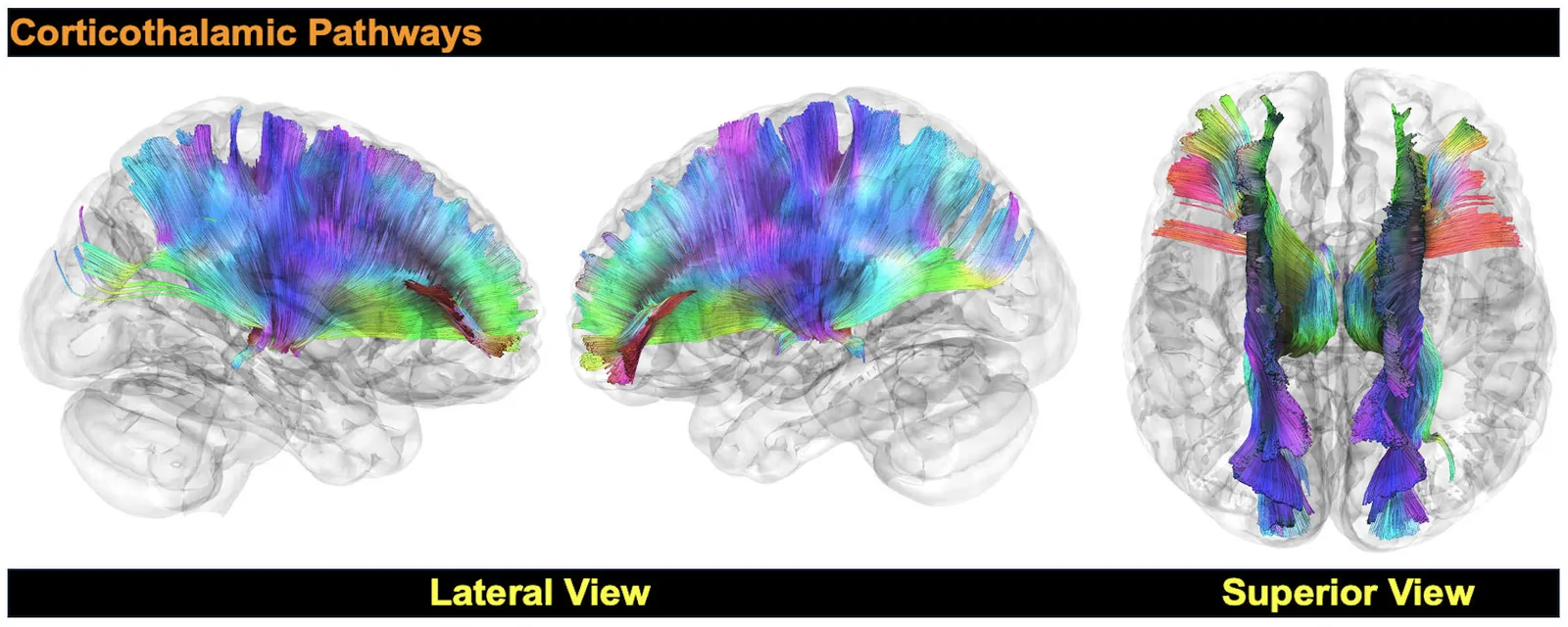
Anatomical localization of key WM contributors for ENET model. Depiction of the anatomical locations of WM pathways (bilateral corticothalamic pathways) identified as key contributors to brain age prediction for ENET model.

**Fig 4:**
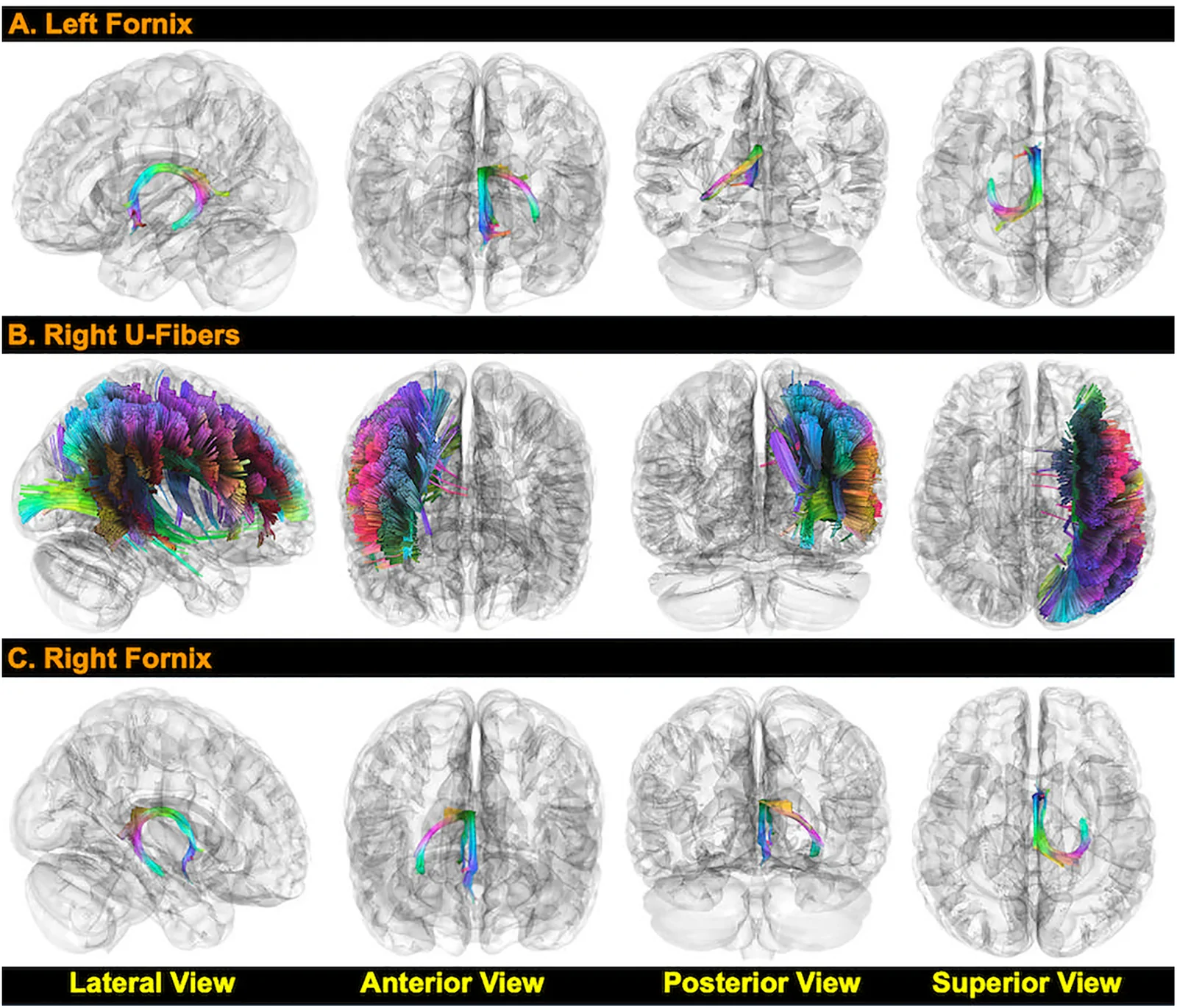
Anatomical localization of key WM contributors for RF model. Depiction of the anatomical locations of WM pathways (bilateral fornix and right U-fibers) identified as key contributors to brain age prediction for RF model.

### Symptom burden as a moderator of the association between ALPS and BAG

Detailed statistics for significant effects for each of the BAG models are reported in Table 3A-B.

**Table 3:** Moderation analyses examining the effects of ALPS index (X) and symptom burden (M) on (A) ENET-based BAG and (B) RF-based BAG, and on (C) left fornix QA in the RF-based brain age model.

Variables	B	SE	t	p	Interpretation
**Moderation analysis predicting BAG from ALPS index (X) and symptom burden (M)**					
**A. ENET-Based BAG**					
Moderation Analysis 1: ALPS Index (Predictor, X) and HN-specific Symptom Severity (Moderator, M)					
Model Fit	R² = .10, F(6,125) = 2.21, p = .05, ΔR² = .051, p = .009				
Predictor (X)	−13.99	10.84	−1.29	.20	–
Moderator (M)	−1.87	0.75	−2.48	.01	–
X × M (Moderation)	13.61	5.12	2.66	.009	–
Conditional Effects	−35.02	14.73	−2.38	.02	Significant when M is 1 SD below the mean
**B. RF-Based BAG**					
Moderation Analysis 1: ALPS Index (Predictor, X) and HN-specific Symptom Severity (Moderator, M)					
Model Fit	R² = .64, F(6,125) = 37.94, p < .001, ΔR² = .029, p = .002				
Predictor (X)	−5.89	4.27	−1.38	.17	–
Moderator (M)	−0.38	0.30	−1.29	.20	–
X × M (Moderation)	6.48	2.02	3.21	.002	–
Conditional Effects	−15.89	5.80	−2.74	.002	Significant when M is 1 SD below the mean
Moderation Analysis 2: ALPS Index (Predictor, X) and Activity-related Symptom Inference (Moderator, M)					
Model Fit	R² = .64, F(6,125) = 36.41, p < .001, ΔR² = .019, p = .012				
Predictor (X)	−7.58	4.58	−1.65	.10	–
Moderator (M)	−0.31	0.22	−1.39	.17	–
X × M (Moderation)	3.46	1.36	2.54	.012	–
Conditional Effects	−12.40	5.61	−2.21	.03	Significant when M is 1 SD below the mean
**C. Moderation analysis predicting the Left Fornix QA from ALPS index (X) and symptom burden (M)**					
Moderation Analysis 1: ALPS Index (Predictor, X) and HN-specific Symptom Severity (Moderator, M)					
Model Fit	R² = .48, F(6,128) = 20.16, p < .001, ΔR² = .024, p = .016				
Predictor (X)	0.090	0.028	3.26	.001	–
Moderator (M)	0.001	0.002	0.54	0.59	–
X × M (Moderation)	−0.032	0.013	−2.44	.016	–
Conditional Effects	0.14	0.037	3.71	< .001	Significant when M is at the mean or below the mean
Moderation Analysis 2: ALPS Index (Predictor, X) and Activity-related Symptom Inference (Moderator, M)					
Model Fit	R² = .50, F(6,128) = 21.47, p < .001, ΔR² = .039, p = .002				
Predictor (X)	0.11	0.029	3.84	.0002	–
Moderator (M)	0.0002	0.001	0.15	.88	–
X × M (Moderation)	−0.027	0.008	−3.15	.002	–
Conditional Effects	0.15	0.035	4.23	<.001	Significant when M is at the mean or below the mean

#### ENET-based BAG.

***Regression Model.*** The omnibus regression model with ENET BAG as the independent measure was not significant for predicting Core Symptom Severity (*F*(6,125) = 1.30, *p* = .26, R^2^ = .06), Activity Symptom Interference (*F*(6,125) = 0.52, *p* = .79, R^2^ = .02), or Affective Symptom Interference (*F*(6,125) = 0.50, *p* = .81, R^2^ = .02). The model was significant at trend-level for HN-specific symptom severity (*F*(6,125) = 2.21, *p* = .05, R^2^ = .10). However, none of these models survived multiple comparison corrections at *p* < .0125 (α = .05/4) to account for multiple comparisons across the four symptom burden domains.

***Moderation.*** The ALPS × BAG interaction was not significant for Core Symptom Severity (*p =* .02), Activity Symptom Interference (*p =* .30), or Affective Symptom Interference (*p =* .32), using a Bonferroni-corrected significance threshold of *p* < .0125 (α = .05/4) to account for multiple comparisons across the four symptom burden domains. However, the ALPS × BAG interaction was significant (*p =* .009) for HN-specific symptom severity, explaining an additional 5.1% of variance beyond main effects and covariates. At lower levels of symptom severity, higher ALPS values were associated with lower BAG (*p =* .02), with attenuation at higher symptom levels.

#### RF-based BAG.

***Regression Model.*** The omnibus regression model with RF BAG as the independent measure was significant for predicting Core Symptom Severity (*F*(6,125) = 34.42, *p* < .0001, R^2^ = .62), HN-specific symptom severity (*F*(6,125) = 37.94, *p* < .0001, R^2^ = .64), Activity Symptom Interference (*F*(6,125) = 36.41, *p* < .0001, R^2^ = .64), and Affective Symptom Interference (*F*(6,125) = 33.63, *p* < .0001, R^2^ = .62). All of these models survived multiple comparison corrections at *p* < .0125 (α = .05/4) to account for multiple comparisons across the four symptom burden domains.

***Moderation.*** The ALPS × BAG interaction was not significant for Core Symptom Severity (*p =* .24) and Affective Symptom Interference (*p =* .36), using a Bonferroni-corrected significance threshold of *p* < .0125 (α = .05/4) to account for multiple comparisons across the four symptom burden domains.

For HN-specific symptom severity, the ALPS × BAG interaction was significant (*p =* .002), explaining an additional 2.9% of variance beyond main effects and covariates. At lower levels of symptom severity, higher ALPS values were associated with lower BAG (*p =* .007), with attenuation at higher symptom levels.

For Activity-related symptom interference, the ALPS × BAG interaction was significant (*p =* .012), explaining an additional 1.8% of variance beyond main effects and covariates. At lower levels of symptom interference, higher ALPS values were associated with lower BAG (*p =* .03), with attenuation at higher interference levels.

Fig 5A and 5B illustrate the significant moderation effects probed at representative low (−1 SD), mean, and high (+1 SD) levels of HN-specific symptom severity and activity-related symptom interference, respectively, consistent with standard moderation analysis practice.

**Fig 5:**
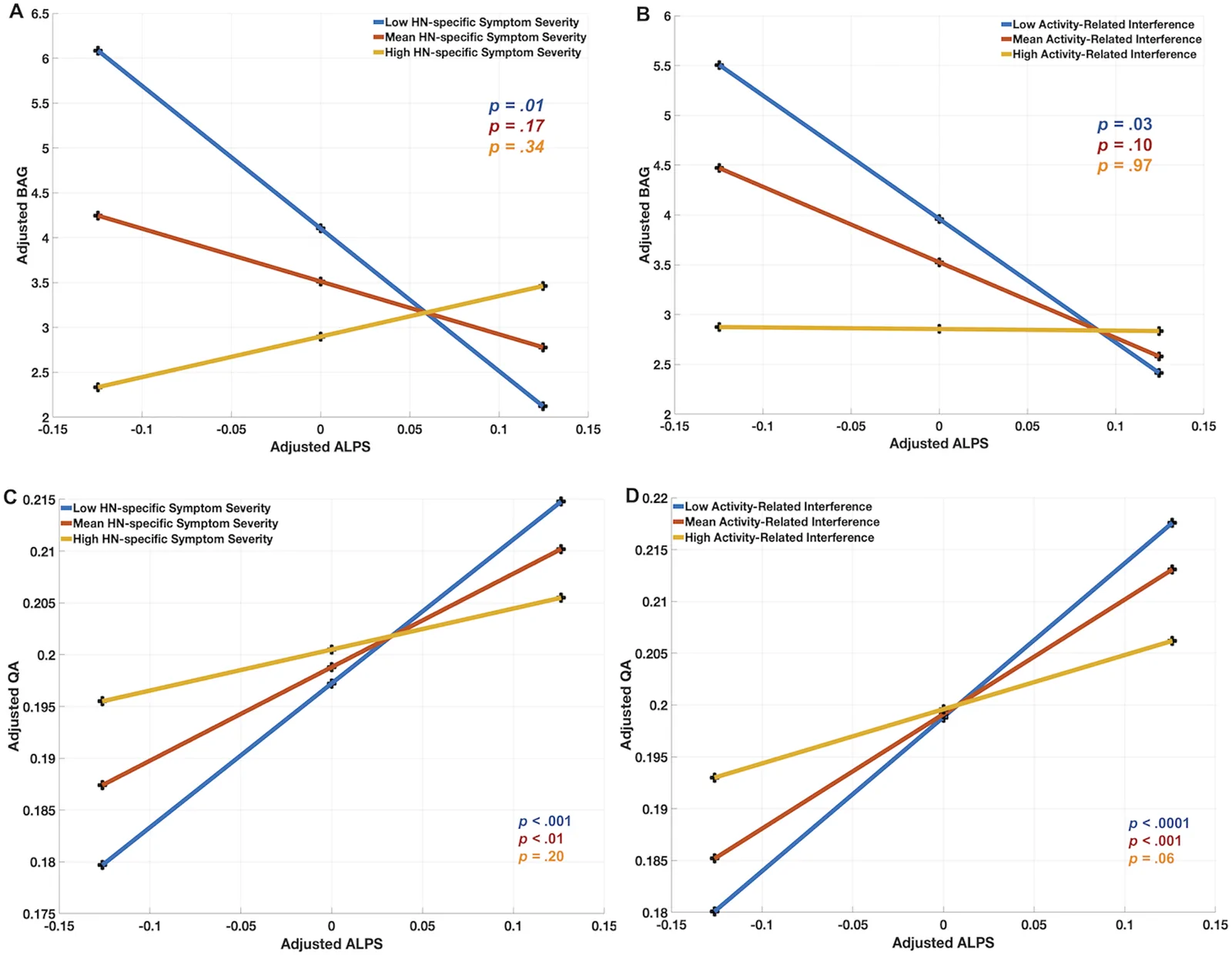
Moderation effects of symptom burden on ALPS–BAG (RF) associations: (A) Significant moderation effect of HN-specific symptom severity on the association of interest, probed at representative low (−1 SD), mean, and high (+1 SD) levels. (B) Significant moderation effect of activity-related symptom interference, similarly, probed at low (−1 SD), mean, and high (+1 SD) levels, consistent with standard moderation analysis practice. Moderation effects with *p*  <  .0125 were considered statistically significant. Moderation effects of multiple symptom domains on ALPS–RF-based QA (left fornix) associations: (C–D) Significant moderation effects probed at representative low (−1 SD), mean, and high (+1 SD) levels of (C) HN-specific symptom severity and (D) activity-related symptom interference, consistent with standard moderation analysis practice. Moderation effects with *p*  <  .017 were considered statistically significant.

### Symptom Burden as a moderator of the association between ALPS and WM microstructure

Detailed statistics for significant effects are reported in Table 3C.

#### ENET-based BAG.

A regression model was conducted for HN-specific symptom severity, as this was the only symptom domain that showed a significant ALPS × symptom burden interaction in the primary ALPS–BAG moderation analysis. A Bonferroni correction was applied across the three key WM bundles identified in the ENET brain age model, yielding a significance threshold of *p* < .017 (α = .05/3). The ALPS × microstructure interactions were not significant after correction for any of the three WM bundles: right corticothalamic pathway QA (*p* = .04), left corticothalamic pathway QA (*p* = .019), or right corticothalamic pathway FA (*p* = .03).

#### RF-based BAG.

Separate regression models were conducted for each symptom domain that showed a significant ALPS × symptom burden interaction in the primary ALPS–BAG moderation analysis. A Bonferroni correction was applied across the three key WM bundles identified in the RF brain age model, yielding a significance threshold of *p* < .017 (α = .05/3).

***HN-Specific Symptom Severity.*** The ALPS × microstructure (for Left Fornix QA) interaction was significant after correction (*p =* .016), explaining an additional 2.4% of variance beyond main effects and covariates. At lower levels of symptom severity, higher ALPS values were associated with greater Left Fornix QA (*p =* .0003), with attenuation at higher symptom levels. No significant interactions were found for Right Fornix QA (F (1,128) = 1.03, *p =* .31) or Right U-Fibers FA (F(1,128) = 1.68, *p =* .20).

***Activity-Related Symptom Interference.*** The ALPS × microstructure (for Left Fornix QA) interaction was significant after correction (*p =* .002), explaining an additional 3.9% of variance beyond main effects and covariates. At lower levels of symptom interference, higher ALPS values were associated with greater Left Fornix QA (*p <* .0001), with attenuation at higher interference levels. No significant interactions were found for Right Fornix QA (F(1,128) = 3.38, *p =* .07) or Right U-Fibers FA (F(1,128) = 0.20, *p =* .65).

Fig 5C–5D illustrate the significant moderation effects probed at representative low (−1 SD), mean, and high (+1 SD) levels of HN-specific symptom severity, and activity-related symptom interference, respectively, consistent with standard moderation analysis practice.

## Discussion

Three principal findings emerged. First, analyses of both brain age models demonstrated model-dependent estimates of brain aging in HN survivors. The ENET model yielded a negative, nonsignificant mean BAG of −1.64 years, whereas the RF model yielded a positive mean BAG of 3.59 years. Given the stronger external generalizability of the RF model in the HN cohort and its greater biological relevance across the ALPS–BAG and ALPS–WM microstructure analyses, the positive RF-derived BAG was interpreted as evidence of accelerated brain aging in HN survivors. Second, accelerated brain aging via RF-based brain age prediction was driven by a distributed set of WM pathways, with the bilateral fornix and right U-fibers emerging as key contributors. Third, symptom burden moderated the associations between ALPS and RF-derived brain age, with the strongest effects observed for HN-specific symptom severity and activity interference. At the feature level, symptom burden also moderated the associations between ALPS and WM microstructure in RF-derived pathways, specifically the left fornix for the same symptom domains (i.e., HN-specific symptom severity and activity interference). Collectively, these findings suggest that the DTI-ALPS index may be associated with more normative brain aging and preserved WM microstructural integrity, particularly among HN survivors with lower symptom burden.

### Brain age prediction and accelerated brain aging in HN survivors

Although the ENET model demonstrated superior predictive performance in the held-out HC test dataset, the RF model demonstrated more robust generalization to the external HN clinical cohort and greater biological relevance in the HN population compared with ENET. This divergence highlights an important methodological consideration: models optimized in large normative datasets do not necessarily transfer effectively to clinical populations characterized by altered or heterogeneous neurobiology. Linear models such as ENET may perform well when brain–age relationships are relatively stable and homogeneous, as in healthy aging. However, clinical populations, particularly cancer survivors, may exhibit non-uniform, regionally specific, or treatment-related alterations that deviate from normative aging trajectories. The superior generalization performance of RF in HN survivors suggests that nonlinear modeling approaches may better capture the complex and potentially non-additive relationships among WM microstructural features in this group. RF models are capable of modeling higher-order interactions and nonlinear feature contributions [56,58] without imposing parametric assumptions. This flexibility may be especially important when predicting brain age in individuals exposed to multimodal cancer treatments, systemic inflammation, vascular changes, or other biological stressors that may differentially affect specific WM pathways.

Using the RF model, HN survivors demonstrated a positive mean BAG of 3.59 years, indicating accelerated brain aging relative to chronological age. This shift is notable given that BAG reflects deviation from normative aging trajectories derived from a large HC sample. Such positive deviations may signal cumulative biological burden, potentially reflecting treatment-related neurotoxicity, systemic inflammatory processes, vascular compromise, or other mechanisms potentially linked to cancer survivorship [4,5,15,18,59–61]. Feature importance analysis further revealed that prediction performance was driven by a distributed set of WM pathways rather than a single dominant tract. Nevertheless, the bilateral fornix (QA) and right U-fibers (FA) emerged as the top contributors following knee-point feature selection. The fornix, a central limbic tract connecting the hippocampus to subcortical structures, is particularly vulnerable to aging and neurodegenerative processes [62–66]. Its prominence in brain age prediction suggests that limbic microstructural integrity may be especially sensitive to cumulative biological stress in HN survivors. The involvement of right U-fibers, which support short-range cortico-cortical connectivity [67–69], further points to the relevance of local association pathways in capturing age-related WM variation.

Collectively, these findings indicate that accelerated brain aging in HN survivors, as reflected by the model demonstrating greater external and biological relevance, is detectable at the level of WM microstructure and may involve distributed but selectively vulnerable pathways, particularly within limbic circuitry. These findings underscore the importance of evaluating complementary modeling approaches and considering both internal predictive performance and external biological relevance when interpreting brain age measures in clinical populations.

### Contextualizing diffusion-based brain age estimation

The predictive performance of the RF model is broadly consistent with previously reported machine learning approaches for brain age estimation, while extending this literature to diffusion-derived neuroimaging measures. A systematic comparison of 27 machine learning models across the HCP, Cambridge Centre for Ageing and Neuroscience (Cam-CAN), and Information eXtraction from Images (IXI) datasets reported MAEs of 2.75–3.12 years, 7.08–10.50 years, and 8.04–9.86 years, respectively, with corresponding correlations between predicted and chronological age of 0.11–0.42, 0.64–0.85, and 0.63–0.79 [70]. In the present study, the RF model achieved R = 0.80, R^2^ = 0.64, MAE = 7.32 years, and RMSE = 9.12 years in an independent HCP test sample (N = 235), placing its performance within the range reported for broader-age datasets such as Cam-CAN and IXI. The relatively higher error compared with models evaluated in the young-adult HCP sample is not unexpected given differences in age distributions, imaging features and modalities, and cross-validation/methodology procedures.

Importantly, direct comparisons across studies should be made cautiously because the prior work primarily used structural morphological measures, whereas the present model was developed using diffusion-derived measures. Brain age prediction based on diffusion imaging features remains relatively uncommon compared with approaches based on morphological measures. Diffusion measures may nevertheless capture complementary aspects of brain aging, including alterations in WM microstructure and tissue organization that are not fully represented by conventional morphometric measures. The present findings therefore provide evidence that diffusion-derived features can support reasonably accurate brain age estimation, with minimal systematic bias in the healthy test sample (mean BAG = 0.54 ± 9.13 years).

Further support for the biological relevance of the model was obtained when it was applied to an external HN cohort. The HN cohort demonstrated evidence of accelerated brain aging, consistent in direction with previous reports of accelerated brain aging following cancer and its treatment [4,5,71–73]. Although most prior cancer-related brain age studies have focused on cancer populations other than HN cancer, the observation of a similar accelerated-aging phenotype in the present HN cancer cohort suggests that diffusion-based BAG may capture clinically relevant alterations in brain aging across cancer populations. Together, these findings support the generalizability and potential clinical utility of diffusion-based brain age estimation while highlighting the complementary information provided by diffusion measures relative to conventional structural morphology.

### Moderation between ALPS and BAG

Moderation effects were domain specific. HN-specific symptom severity and activity-related symptom interference significantly moderated the ALPS–BAG association, whereas core symptom severity and affective interference did not. At lower levels of HN-specific symptoms and activity-related interference, higher ALPS values were associated with lower BAG (i.e., younger and healthy appearing brains). This association attenuated at higher symptom levels. These findings suggest that glymphatic-related processes may confer protection against accelerated brain aging—but primarily among individuals with lower physical and functional symptom burden. HN-specific symptoms likely reflect localized treatment sequelae and cumulative physiological stress, while activity-related interference captures the extent to which symptoms disrupt daily functioning. These domains may reflect systemic inflammation, sleep disruption, metabolic strain, or neurovascular factors, which could plausibly influence both DTI-ALPS measures and WM aging but were not directly assessed in the present study. In contrast, core symptoms represent broader cancer-related complaints and may be less sensitive to neurobiological aging mechanisms specific to HN survivorship. The absence of moderation by affective interference suggests that emotional impact alone may not strongly influence global structural aging metrics such as BAG within this cohort.

### Moderation between ALPS and WM microstructure

At the tract level, moderation effects were again pronounced and extended across HN-specific and activity-related domains. Both of these domains significantly moderated the association between ALPS and Left Fornix QA. Across these two identified domains, a consistent pattern emerged: at lower levels of symptom burden, higher ALPS values were associated with greater Left Fornix QA, with attenuation at higher symptom levels. The broader pattern of moderation at the tract level (and with BAG) suggests an important interplay between limbic microstructure, glymphatic-related processes, and symptom burden [74–79]. HN-specific symptoms reinforced the possibility that disease-specific symptom reports may be tightly coupled to limbic microstructural vulnerability. Activity-related interference captures the degree to which symptoms disrupt daily functioning, potentially indexing cumulative stress exposure and neuroendocrine burden. Notably, no significant moderation effects were observed for Right Fornix QA or Right U-Fibers FA. The lateralized association indicates potential hemispheric specificity, whereby the left fornix is particularly influenced by glymphatic-related processes in the context of symptom burden. This pattern may reflect asymmetric contributions of brain structure to brain age estimation and differential susceptibility of hemispheres to aging-related processes [80,81].

Taken together, these findings support a model in which glymphatic-related processes are associated with more favorable brain aging and limbic WM integrity – but this association is conditional upon symptom burden. Lower levels of HN-specific and functionally disruptive symptoms appear to permit a strong coupling between glymphatic dynamics and structural brain health. As symptom burden increases, this coupling weakens, potentially reflecting cumulative biological stress that overwhelms compensatory mechanisms. The convergence of moderation effects on Left Fornix QA and BAG underscores the central role of limbic circuitry in linking glymphatic-related processes, symptom experience, and structural brain aging in HN survivors. Moreover, the stronger and more consistent effects at the tract level suggest that microstructural measures may detect biologically meaningful interactions that are partially diluted in global aging indices.

### Clinical implications

The observed atypical brain aging patterns may provide a quantitative marker for identifying HN cancer survivors with greater-than-expected alterations in brain health who could benefit from closer symptom monitoring. Integrating BAG with WM integrity, DTI-ALPS, and patient-reported symptom burden may help characterize individual differences in survivorship-related brain health and support more personalized survivorship care. In practice, such information could facilitate earlier referral for symptom assessment, behavioral or supportive interventions, and longitudinal monitoring in patients showing greater vulnerability. Prospective studies are needed to determine whether these measures can improve risk stratification and patient outcomes and to establish their utility for guiding clinical interventions.

### Limitations and future directions

The cross-sectional design limits causal inference among the DTI-ALPS index, brain aging, and brain health, and the observed associations may reflect shared effects of cancer, treatment, or systemic factors. Although we adjusted for age, sex, and MRI–MDASI interval, tumor characteristics, treatment exposure, and vascular/metabolic comorbidities were not comprehensively accounted for because some variables were incompletely available or highly interrelated. Additionally, the substantial interval between MRI and MDASI assessment limits temporal alignment; adjustment for this interval cannot establish contemporaneous or causal associations. Thus, residual confounding and temporal uncertainty cannot be excluded. Future longitudinal studies with repeated imaging and symptom assessments, comprehensive clinical characterization, and closely timed assessments are warranted. The ALPS index provides only an indirect measure of glymphatic-related processes, and symptom burden was self-reported, potentially influenced by contextual factors. Symptom severity in this cohort was generally mild, limiting variability, though associations with imaging measures were still detected. Finally, the focus on global symptom indices may have obscured symptom-specific relationships, which future studies should explore.

## Conclusions

Our findings suggest that symptom burden may condition the associations between glymphatic-related processes, brain aging, and WM integrity in HN survivors. Although evidence for accelerated brain aging was not fully consistent across the linear ENET and nonlinear RF models, the RF model demonstrated stronger external generalizability and greater biological relevance in the HN cohort; therefore, the observed positive BAG in the RF model was interpreted as evidence of accelerated brain aging in this population. The results highlight an interaction among glymphatic-related processes, limbic WM microstructure, brain aging, and symptom burden, providing insight into potential mechanisms underlying variability in neurobiological aging. Longitudinal and mechanistic studies are warranted to determine the temporal and biological relationships among these factors.

## Supporting information

S1 File Tumor and treatment characteristics of HN participants. (DOCX)
