# Supplementary material for: Lower symptom burden strengthens the association between DTI-ALPS index and brain aging in head and neck cancer survivors
Source: PLoS One. 2026 Nov 13;21(11):e0360924. doi: 10.1371/journal.pone.0360924 (PMC13766262; doi:10.1371/journal.pone.0360924)
Supplement: Supplementary file 1 [file pone.0360924.s001.docx]

**Supplementary Section**

**S1. HN Tumor and Treatment Characteristics**

***S1a. Tumor Characteristics:*** Primary tumor type and anatomical location were heterogeneous across participants, reflecting real-world clinical presentations in HN cancer survivorship. The majority of participants had **mucosal HN squamous cell carcinoma** (54.8%), involving the larynx, oropharynx, oral cavity, and nasopharynx/pharynx. **Cutaneous malignancies**, including cutaneous squamous cell carcinoma (6.7%) and melanoma (primary and metastatic combined; 11.1%), comprised a substantial proportion of cases, predominantly affecting the skin, face, and scalp regions. Additional tumor types included **salivary gland (parotid) malignancies** (4.4%) and other less common tumors involving the mandible, sinus, retromolar region, and hypopharynx (3.7%). Nearly one-fifth of the cohort (19.3%) was categorized under **personal history codes**, reflecting prior HN cancer diagnoses with historical primary sites that may not correspond to active treatment fields at the time of MRI acquisition. Primary tumor locations most frequently involved the **oropharynx** (20.7%), **oral cavity** (15.6%), **larynx** (11.1%), and **skin/face/scalp regions** (17.8%), underscoring the anatomical heterogeneity of the survivor cohort. Disease stage at diagnosis spanned **Stages I–IV**, capturing a broad clinical spectrum.

***S1b. Treatment Characteristics:*** Treatment exposure among the 135 HN cancer survivors was heterogeneous, spanning single-modality, dual-modality, and multimodal regimens consistent with contemporary standard-of-care. Dual-modality approaches were most common, with **chemotherapy plus radiation therapy** representing the largest subgroup (21.5%), followed by **surgery with adjuvant radiation** (11.9%). Single-modality treatments were also frequent, including **surgery alone** (17.8%) and **radiation therapy alone** (10.4%). More intensive **trimodality regimens** combining surgery, chemotherapy, and radiation were administered to 7.4% of participants, while an additional subset received **immunotherapy-containing regimens**, most commonly chemotherapy plus immunotherapy with radiation (5.9%). Less prevalent treatment patterns included chemotherapy with surgery, chemotherapy alone, other surgical interventions, immunotherapy-based dual therapies, and cases with no documented treatment (each ≤3.7%). A small number of participants received rare or specialized combinations such as proton therapy, endocrine therapy, or hyperthermia in conjunction with standard modalities. Collectively, this broad spectrum of treatment exposures reflects real-world clinical practice in HN cancer survivorship. Retaining this heterogeneity enhances the ecological validity and generalizability of downstream neuroimaging and brain age analyses.
