# Supplementary material for: Lower symptom burden strengthens the association between DTI-ALPS index and brain aging in head and neck cancer survivors
Source: PLoS One. 2026 Nov 13;21(11):e0360924. doi: 10.1371/journal.pone.0360924 (PMC13766262; doi:10.1371/journal.pone.0360924)
Supplement: Supplementary file 2 [file pone.0360924.s003.docx]

**Response to Reviewers Comments**

**Manuscript ID:** PONE-D-26-25905

**Original Title: Lower symptom burden strengthens the association between glymphatic function and brain aging in head and neck cancer survivors**

**Revised Title: Lower symptom burden strengthens the association between DTI-ALPS index and brain aging in head and neck cancer survivors**

***Reviewer #1:*** *This study integrates diffusion MRI–based brain age prediction, the DTI-ALPS index, white matter tract microstructure, and symptom burden to investigate brain aging characteristics and their potential influencing factors in long-term survivors of head and neck cancer. The research question has clinical relevance, the normative healthy cohort is relatively large, and the authors employed nested cross-validation, bias correction, and moderation analyses. The overall study design is somewhat innovative. However, the manuscript still has the following issues.*

**Response:** We sincerely thank the reviewer for the positive and thoughtful assessment of our study. We agree that few aspects of the manuscript require further clarification and improvement. In response to the reviewer’s comments, we have carefully revised the manuscript. Detailed responses to each of the reviewer’s concerns are provided below, with corresponding changes highlighted in the revised manuscript.

***Reviewer Comment 1.*** *The DTI-ALPS index should not be directly equated with glymphatic system function or operational efficiency. The manuscript repeatedly uses terms such as “glymphatic function,” “glymphatic efficiency,” and “glymphatic clearance” to describe the meaning of ALPS, and states that DTI-ALPS can noninvasively quantify glymphatic system efficiency in vivo. In fact, DTI-ALPS is an indirect diffusion MRI metric calculated from directional diffusivities in specific periventricular white matter regions. The ALPS index may also be influenced by local white matter fiber architecture, brain atrophy, ventricular enlargement, white matter lesions, ROI placement, partial-volume effects, and diffusion acquisition parameters. The manuscript currently infers that the glymphatic system has a neuroprotective effect from associations of the ALPS index with BAG or fornix QA. This biological interpretation clearly exceeds what can be supported by this metric and by a cross-sectional study.*

**Response:** We thank the reviewer for this important clarification. We agree that the DTI-ALPS index is an indirect diffusion MRI–based measure and should not be interpreted as a direct measure of glymphatic system function, efficiency, or clearance. We have therefore substantially revised the manuscript to ensure that our terminology and biological interpretations do not overstate what can be inferred from the ALPS index. Specifically, throughout the manuscript (including title), we have replaced definitive terms such as “glymphatic function,” “glymphatic efficiency,” and “glymphatic clearance” with more appropriate terminology, including “DTI-ALPS index” or “ALPS-derived measure,” where appropriate. We have also removed statements suggesting that DTI-ALPS can directly quantify glymphatic system efficiency in vivo. In addition, we explicitly acknowledge that the ALPS index may be influenced by factors beyond glymphatic-related processes, including local white matter fiber architecture, brain atrophy and ventricular enlargement, white matter lesions, ROI placement, partial-volume effects, and diffusion acquisition parameters. Importantly, we have revised our interpretation of the associations between ALPS and brain age gap or fornix QA. We no longer describe these associations as evidence that the glymphatic system has a neuroprotective effect. Instead, we interpret them as associations between an ALPS-derived diffusion metric and neuroimaging/symptom-related measures. Given the cross-sectional nature of the study, we also acknowledge that causal or mechanistic conclusions regarding glymphatic function, neuroprotection, or brain aging cannot be established.

These revisions have been made throughout the Abstract, Introduction, Material and Methods, Results, Discussion, and Limitations and Conclusion to ensure consistency and to appropriately frame the DTI-ALPS index as an indirect and potentially nonspecific diffusion MRI measure rather than a direct measurement of glymphatic function.

***Reviewer Comment 2.*** *The patient cohort is defined by the authors as an external validation cohort. However, the final model was selected after comparing model performance in this cohort. Therefore, the patient data were effectively involved in model selection and no longer constitute a fully independent external test dataset.*

**Response:** We thank the reviewer for this important clarification. We agree that consideration of patient-cohort performance during final model selection means that the cohort should not be considered a completely untouched independent test set. However, the patient cohort was not used to train the models or estimate model parameters. Rather, we evaluated the candidate models in the patient cohort to identify the model that demonstrated the **best generalization and the least systematic bias** when transferred from the healthy control population to the head and neck cancer population. Model selection was therefore based on the overall generalizability and bias of the candidate models (lines 273-274 and lines 277-284) rather than on statistical significance or the magnitude of the observed patient effect. We have clarified this distinction in the revised manuscript and now refer to the patient cohort as an **external evaluation/validation cohort (line 273)**, rather than a fully independent test set. We have also acknowledged that using patient-cohort performance to select the final model introduces a degree of data dependence and therefore limits the interpretation of the patient cohort as an unbiased final test dataset. Importantly, the selected model was not retrained using patient data, preserving its application as an externally derived model to the cancer cohort (lines 277-284).

***Reviewer Comment 3.*** *Key clinical confounders were not included in the models. ALPS, white matter diffusion metrics, BAG, and symptom burden may all be influenced by tumor site, stage and HPV status, radiation dose, irradiation field and brain radiation exposure, hypertension, diabetes, dyslipidemia, and cardiovascular or cerebrovascular disease. The manuscript adjusts only for age, sex, and the MRI–MDASI interval, which is insufficient to exclude these shared causes. In particular, the authors repeatedly interpret the findings in terms of inflammatory, sleep-related, and vascular mechanisms, although these factors were neither directly measured nor controlled.*

**Response:** We thank the reviewer for this important comment. We agree that tumor characteristics, treatment exposure, and vascular/metabolic comorbidities may influence the observed neuroimaging and symptom measures. Our models were intentionally parsimonious and included prespecified covariates (age, sex, and MRI–MDASI interval) that were consistently available and directly relevant to the primary analyses. We did not include all potential clinical variables because several were incompletely available across participants, while others were highly interrelated (e.g., tumor characteristics and treatment variables). Including numerous correlated or incompletely measured variables could substantially reduce the analyzable sample, introduce selection bias, and result in unstable or potentially over-adjusted models. Moreover, the objective of this study was to characterize associations rather than estimate independent causal effects of specific tumor or treatment factors.

More specifically, we believe factors such as tumor characteristics, cancer stage, and treatment modality function more plausibly as mediators than confounders in this design — treatment and tumor burden likely lie on the causal pathway between HN cancer and brain-age deviation, rather than being independent common causes, and adjusting for them risks overadjustment bias that would mask the very effect we aim to characterize. In addition, with n = 135, several treatment subgroups contain only 5–10 participants (see Supplementary section S1), and tumor site/stage strongly predict treatment selection, so entering these as covariates would introduce collinearity and unstable estimates rather than improve confound control. Finally, our study's aim was to characterize brain-age deviation across the real-world heterogeneity of HN cancer survivorship rather than to isolate a treatment-independent effect and adjusting for treatment would work against this goal and reduce generalizability.

We acknowledge that this approach does not eliminate residual confounding. We have therefore added this limitation to the revised manuscript (lines 464-471) and substantially tempered our interpretation of inflammatory, sleep-related, vascular, and other potential mechanisms, which were not directly measured in this study (lines 422-424).

***Reviewer Comment 4.*** *A positive mean BAG alone is insufficient to demonstrate accelerated brain aging in the patient group. The authors mainly infer accelerated brain aging from the mean BAG of 3.59 years generated by the random forest model in the patient group. However, the manuscript should further provide a formal test of whether BAG differs from zero, together with the corresponding 95% confidence interval.*

**Response:** We thank the reviewer for this important point. We agree that a positive mean BAG alone does not establish accelerated brain aging at the group level. We have therefore performed a formal one-sample test of BAG against zero in the patient cohort and now report the corresponding 95% confidence interval. The mean BAG was **3.59 (SD = 8.56) years (95% CI: [2.11, 5.06])**, one-sample t-test: **t[131] = 4.82, p < .001)**, indicating that the mean BAG was significantly greater than zero. These results have been added to the revised Results (lines 278-280).

***Reviewer Comment 5.*** *The Methods section lacks key information required to ensure reproducibility.*

**Response:** We thank the reviewer for highlighting this important issue. We agree that additional methodological details are necessary to ensure transparency and reproducibility. We have therefore carefully revised and expanded the Methods section to provide greater detail regarding participant selection (lines 126-128, 130-131), MRI preprocessing (lines 165-175), diffusion-derived measures (lines 176-188) and age harmonization for brain age prediction model (lines 217-223), and specific covariate selection (lines 464-471). We have also clarified the relevant quality-control procedures (lines 165-175), and analysis parameters (lines 179-185). These revisions are intended to provide sufficient methodological detail to facilitate independent replication of the study.

***Reviewer Comment 6.*** *There is a substantial temporal mismatch between MRI and symptom assessment. The MDASI evaluates symptoms during the preceding 24 hours, whereas the mean interval between MRI and MDASI assessment was 1.76 ± 4.83 years. Including the interval as a covariate does not resolve the lack of temporal alignment between the two measurements.*

**Response:** We thank the reviewer for highlighting this important limitation. Although we included the MRI–MDASI interval as a covariate to account for this temporal difference, we acknowledge that this adjustment cannot fully establish temporal alignment or eliminate the possibility that brain measures and symptom burden changed during the intervening period. We have therefore revised the manuscript to explicitly acknowledge the temporal mismatch as a limitation and have tempered our interpretation of the associations between neuroimaging measures and symptom burden (lines 464-471). We now emphasize that these findings should be interpreted as associations rather than evidence of contemporaneous or causal relationships. Future prospective studies with MRI and symptom assessments performed within a closely aligned time window will be important to confirm these findings.

**Reviewer #2:** *In this manuscript Bajaj et al have tested machine learning algorithms to test if survivors of head and neck cancer have accelerated brain aging and whether glymphatic function influences brain aging. They report accelerated brain aging and disruption of limbic white matter pathways in HN cancer survivors while glymphatic system function may have a protective effect. The study addresses an important gap in oncology research by investigating the impact of cancer therapy on brain aging. The manuscript is well written but could benefit from minor revisions. Below are some specific comments and suggestions.*

**Response:** We thank the reviewer for the positive and constructive assessment of our manuscript. In response to the reviewer’s suggestions, we have carefully revised the manuscript. We address each of the specific comments below.

***Reviewer Comment 1.*** *The authors should describe the method used for age range harmonization.*

**Response:** We thank the reviewer for this suggestion. To harmonize the age ranges between the healthy control and HN cancer cohort, we used an age-range restriction approach rather than resampling or individual age matching. First, HN cancer participants outside the minimum-to-maximum age range of the healthy control cohort were excluded, reducing the cancer cohort from **135 to 132 participants**. We then restricted the healthy control cohort to participants whose ages were within the resulting age range of the HN cohort, reducing the healthy control cohort from **1,228 to 1,176 participants**. Thus, both cohorts were analyzed within a common overlapping age range, minimizing extrapolation of the brain age model beyond the age distribution represented in the study cohorts. This procedure has been clarified in the revised Methods section (lines 217-223).

***Reviewer Comment 2.*** *The authors should discuss the clinical implications of their observations and how it can help improve patient care.*

**Response:** We thank the reviewer for this important comment. We have expanded the Discussion (lines 454-462) to clarify how our findings may contribute to patient care. The observed accelerated brain aging could potentially help identify HN cancer survivors with greater-than-expected alterations in brain health who may benefit from closer symptom monitoring. Incorporating brain age gap (BAG) with white matter measures and patient-reported symptom burden may further support individualized survivorship assessment and identification of patients who may benefit from targeted supportive or rehabilitative interventions. The association between higher DTI-ALPS index and lower BAG also provides a potential imaging marker for monitoring brain health. Although these applications require prospective validation, this framework could ultimately facilitate earlier identification of vulnerable survivors and more personalized survivorship care.

***Reviewer Comment 3.*** The authors should discuss how the present brain age prediction model compares with previously published models in terms of methodology and predictive performance.

**Response:** We thank the reviewer for this important comment. We have expanded the Discussion to contextualize the methodology and predictive performance of our brain age model relative to previously published machine learning approaches. In particular, we now compare our most generalizable model (i.e., random forest (RF) model) with a systematic evaluation of 27 machine learning models across the Human Connectome Project (HCP), Cambridge Centre for Ageing and Neuroscience (Cam-CAN), and Information eXtraction from Images (IXI) datasets. While that prior work focused primarily on structural morphological measures, our model was developed using diffusion-derived measures. Brain age prediction using diffusion imaging features has been comparatively rarely investigated, despite diffusion measures providing complementary information about age-related alterations in white matter and tissue microstructure.

Our RF model demonstrated good generalization to an independent, unseen HCP test sample, with R=0.80, R^2^=0.64, MAE=7.32 years, and RMSE=9.12 years. Although the MAE was higher than that reported for models evaluated in the young-adult HCP sample in the prior study, it falls within the performance range reported for the broader-age Cam-CAN and IXI datasets. Direct comparisons should nevertheless be interpreted cautiously because of differences in imaging modality, age distributions, feature sets, and cross-validation/methodology procedures.

We further evaluated the model in an independent cancer cohort and observed evidence of accelerated brain aging in patients with HN cancer. The direction and magnitude of the observed accelerated-aging phenotype were broadly consistent with previous brain-age studies in cancer populations, despite the fact that much of the existing literature has focused on other cancer types rather than just head and neck cancer. Thus, the convergence of good predictive performance in an independent healthy test sample and biologically plausible accelerated-aging estimates in an external cancer cohort provides additional support for the utility of diffusion-based brain age modeling. These comparisons and considerations have now been incorporated into the revised Discussion (lines 387–413).

***Reviewer Comment 4.*** Did the authors evaluate whether treatment modality, treatment duration, or cancer stage were associated with brain age gap or white matter microstructural measures?

**Response:** We thank the reviewer for this important question. Treatment modality, treatment duration, and cancer stage were not included as primary covariates in the brain age and white matter analyses because the study was designed primarily to characterize overall neuroimaging alterations in *long-term* head and neck cancer survivors rather than to evaluate treatment-specific effects. In addition, treatment-related variables were not uniformly available or sufficiently detailed across the cohort, and several treatment characteristics were highly interrelated with significant overlaps (see Supplementary section S1). We acknowledge that these factors may contribute to variability in BAG and white matter microstructure and have therefore added this issue as a limitation (lines 464-471). We have also tempered our interpretation to avoid attributing the observed neuroimaging alterations specifically to cancer treatment or disease stage. Future studies with comprehensive treatment and disease-characterization data will be needed to determine treatment- and stage-specific effects.

***Reviewer Comment 5.*** Does the apparent protective association of glymphatic function diminish with increasing symptom burden, or does reduced glymphatic function simply reflect the extent of underlying brain injury, thereby serving as a marker rather than a mediator of symptom severity? The authors should comment on this.

**Response:** We thank the reviewer for this insightful comment. We agree that the observed associations cannot distinguish whether the DTI-ALPS index represents a protective factor, a marker of underlying brain injury, or a correlate of symptom burden. Because our study is cross-sectional and DTI-ALPS is an indirect diffusion MRI measure, we cannot establish mediation or directionality. Our moderation analyses should not be interpreted as evidence of protective association. Reduced ALPS may alternatively reflect broader white matter or brain alterations associated with greater symptom burden. We have therefore revised our manuscript thoroughly to explicitly acknowledge these alternative interpretations and now describe the ALPS-related findings as **moderating associations** rather than evidence of a protective or mediating glymphatic effect. Longitudinal studies with repeated imaging and symptom assessments will be necessary to determine the temporal and causal relationships among ALPS, brain injury, and symptom burden.

***Reviewer Comment 6.*** The authors should indicate whether the model, code, or feature extraction pipeline are publicly available.

**Response:** We thank the reviewer for this suggestion. The study-specific code, model, and feature-extraction pipeline are not currently publicly available because the analyses were developed and executed within our institution using clinical research data subject to institutional data-use, privacy, and governance requirements. In particular, the underlying clinical and neuroimaging data cannot be publicly distributed, and the associated analysis pipeline contains dataset-specific processing and feature-extraction procedures. We have clarified this in the revised Data Availability statement that de-identified data, appropriate analysis code and documentation may be made available from the University of Texas MD Anderson Cancer Center and the corresponding author only upon reasonable request, subject to institutional policies and applicable data-use restrictions.
