# Supplementary material for: Lower symptom burden strengthens the association between DTI-ALPS index and brain aging in head and neck cancer survivors
Source: PLoS One. 2026 Nov 13;21(11):e0360924. doi: 10.1371/journal.pone.0360924 (PMC13766262; doi:10.1371/journal.pone.0360924)
Supplement: Supplementary file 3 [file pone.0360924.s004.docx]

**Response to Reviewers Comments**

**Manuscript ID:** PONE-D-26-25905

**Title: Lower symptom burden strengthens the association between DTI-ALPS index and brain aging in head and neck cancer survivors**

***Reviewer #1***

***Comment 1A:*** *The authors have made substantial efforts to address the comments raised in the previous round. In particular, the terminology surrounding the DTI-ALPS index has been revised, additional methodological details have been provided, and a formal statistical test of whether the mean brain age gap (BAG) differed from zero has been added. These revisions have improved the manuscript.*

**Response:** We sincerely thank the reviewer for the positive assessment of our revised version.

***Comment 1B:*** However, the following issue remains unresolved. Although the authors now acknowledge that the patient cohort contributed to final model selection and have redefined it as an external evaluation/validation cohort, this revision does not resolve the data dependence introduced by the model-selection procedure. Of particular concern, ENET showed better predictive performance than RF in the independent held-out HC test set (R² = 0.76, MAE = 5.80 years vs. R² = 0.64, MAE = 7.32 years), whereas the two models produced BAG estimates in opposite directions when applied to the same HN cancer cohort: the mean BAG was −1.64 years for ENET but +3.59 years for RF. The authors subsequently selected RF on the basis of its generalizability and systematic bias in the patient cohort and then used the positive BAG generated by RF to support the central conclusion that “HN survivors exhibit accelerated brain aging”; the revised manuscript also explicitly acknowledges that patient-cohort performance was considered during final model selection. Therefore, the issue is no longer merely whether this cohort can be considered an independent external test set; rather, the central conclusion of accelerated brain aging appears to be materially dependent on the choice of prediction model. The authors’ statement that RF was not retrained using patient data does not eliminate this post-selection bias. The final model should ideally be selected using criteria completely independent of the HN cohort, for example, based only on the HC training data, nested cross-validation, and the held-out HC test set, and then applied once to the HN cohort after model selection has been finalized. Alternatively, the authors should report the ENET and RF findings in parallel and explicitly acknowledge that the conclusion of accelerated brain aging is not robust to model choice. Under the current analytical framework, the positive BAG obtained from an RF model selected after evaluating its performance in the patient cohort does not provide sufficiently independent evidence to support the conclusion of accelerated brain aging.

**Response:** We thank the reviewer for raising this important concern regarding model selection and post-selection dependence. We greatly appreciate the reviewer’s careful consideration of this issue, which prompted us to re-examine our analytical framework and substantially clarify how the two brain age models are interpreted. We agree with the fact that the RF model was originally considered for subsequent analyses based partly on its performance in the HN cohort introduces data dependence and that the fact that the model was not retrained using patient data does not, by itself, eliminate this concern. Following the reviewer’s recommendation, we have therefore revised the analysis and manuscript to report the ENET and RF models in parallel rather than treating the HN cohort as an independent dataset for selecting a single final model. We now present the predictive performance and BAG estimates from both models, as well as their respective associations with ALPS and symptom burden.

As reported before, ENET showed superior predictive performance in the held-out HC test set, whereas RF showed stronger generalization in the external HN cohort compared with. The models also yielded different BAG estimates in the HN cohort: ENET produced a negative, nonsignificant BAG, whereas RF produced a positive, significant BAG. In the revised manuscript. we further examined whether these model-dependent BAG estimates showed convergent biological relationships with the ALPS index. Notably, the ENET-derived BAG did not demonstrate a significant association with ALPS in the omnibus regression models for any of the four symptom-burden domains after correction for multiple comparisons, nor were the ALPS × symptom-burden interactions significant in the corresponding moderation analyses. In contrast, the RF-derived BAG demonstrated significant ALPS associations in the omnibus regression analyses across all four symptom-burden domains after multiple-comparison correction, with significant ALPS × symptom-burden interactions observed for two of the four domains after correction. Feature-level analyses further demonstrated biologically coherent ALPS–WM microstructure relationships involving WM pathways identified as key contributors to the RF brain age model.

Based on these parallel analyses, we have revised our interpretation to distinguish **predictive model performance from biological relevance in the target clinical population**. We do not claim that RF is universally superior to ENET, nor do we use the HN cohort to retrospectively establish RF as the sole optimal prediction model. Rather, the two models were carried forward because they provide complementary information: ENET, a predominantly linear model, showed stronger internal predictive performance in held-out healthy controls, whereas RF, which can capture nonlinear and higher-order relationships, demonstrated stronger external generalizability and greater biological relevance in the HN cohort. The consistency of the RF-derived BAG with the ALPS and symptom-related findings provided the basis for interpreting the positive RF BAG as evidence of accelerated brain aging in the HN cohort.

We have also explicitly tempered the conclusion in the revised manuscript. Specifically, we now acknowledge that the direction and magnitude of BAG were not fully consistent across the two modeling approaches and that the evidence for accelerated brain aging is therefore **model-dependent rather than uniformly robust across models**. The conclusion of accelerated brain aging is based on the model that demonstrated stronger generalization to the clinical population and greater biological relevance within the HN cohort, while the ENET findings are reported transparently rather than being omitted or treated as a failed model. We believe this revised approach more appropriately reflects the uncertainty associated with applying brain age models developed in healthy populations to clinical cohorts and avoids presenting the RF-derived BAG as an independently validated finding.

In the revised manuscript, we now report both Bonferroni corrected ENET and RF results throughout the relevant Results and Discussion sections and explicitly discusses the implications of model choice, external generalizability, and the differing functional forms of the two algorithms. **All new results, corresponding text, and related edits throughout the manuscript have been highlighted in the revised version. The relevant tables have also been revised to include the ENET results, and new figures have been added to present the ENET brain age model results and associated findings.** These changes provide a transparent parallel presentation of both modeling approaches and directly address the reviewer’s concern regarding model-selection dependence.
