# Supplementary material for: Lower symptom burden strengthens the association between DTI-ALPS index and brain aging in head and neck cancer survivors
Source: PLoS One. 2026 Nov 13;21(11):e0360924. doi: 10.1371/journal.pone.0360924 (PMC13766262; doi:10.1371/journal.pone.0360924)
Supplement: Peer review history

# Author comment: Author response to Decision Letter 0

**DOI:** 10.1371/journal.pone.0360924.r001

**Transfer Alert**

This paper was transferred from another journal. As a result, its full editorial history (including decision letters, peer reviews and author responses) may not be present.

5 Jun 2026

# Peer review: Decision Letter 0

**DOI:** 10.1371/journal.pone.0360924.r002

**Authors:** Feng Liu

20 Jul 2026

Dear Dr. Bajaj,

Thank you for submitting your manuscript to PLOS ONE. After careful consideration, we feel that it has merit but does not fully meet PLOS ONE’s publication criteria as it currently stands. Therefore, we invite you to submit a revised version of the manuscript that addresses the points raised during the review process.

Please submit your revised manuscript by Sep 18 2026 11:59PM. If you will need more time than this to complete your revisions, please reply to this message or contact the journal office at plosone@plos.org. When you're ready to submit your revision, log on to https://www.editorialmanager.com/pone/ and select the 'Submissions Needing Revision' folder to locate your manuscript file.

- A letter that responds to each point raised by the academic editor and reviewer(s). You should upload this letter as a separate file labeled 'Response to Reviewers'.
- A marked-up copy of your manuscript that highlights changes made to the original version. You should upload this as a separate file labeled 'Revised Manuscript with Track Changes'.
- An unmarked version of your revised paper without tracked changes. You should upload this as a separate file labeled 'Manuscript'.

If you would like to make changes to your financial disclosure, please include your updated statement in your cover letter. Guidelines for resubmitting your figure files are available below the reviewer comments at the end of this letter.

If applicable, we recommend that you deposit your laboratory protocols in protocols.io to enhance the reproducibility of your results. Protocols.io assigns your protocol its own identifier (DOI) so that it can be cited independently in the future. For instructions see: https://journals.plos.org/plosone/s/submission-guidelines#loc-laboratory-protocols. Additionally, PLOS ONE offers an option for publishing peer-reviewed Lab Protocol articles, which describe protocols hosted on protocols.io. Read more information on sharing protocols at https://plos.org/protocols?utm_medium=editorial-email&utm_source=authorletters&utm_campaign=protocols.

As the corresponding author, your ORCID iD is verified in the submission system and will appear in the published article. PLOS supports the use of ORCID, and we encourage all coauthors to register for an ORCID iD and use it as well. Please encourage your coauthors to verify their ORCID iD within the submission system before final acceptance, as unverified ORCID iDs will not appear in the published article. *Only*  the individual author can complete the verification step; PLOS staff *cannot*  verify ORCID iDs on behalf of authors.

We look forward to receiving your revised manuscript.

Kind regards,

Feng Liu, Ph.D.

Academic Editor

PLOS One

Journal Requirements:

When submitting your revision, we need you to address these additional requirements.

1. Please ensure that your manuscript meets PLOS ONE's style requirements, including those for file naming. The PLOS ONE style templates can be found at

https://journals.plos.org/plosone/s/file?id=wjVg/PLOSOne_formatting_sample_main_body.pdf and

https://journals.plos.org/plosone/s/file?id=ba62/PLOSOne_formatting_sample_title_authors_affiliations.pdf

2. Thank you for stating the following financial disclosure: [This work was supported by the Cancer Prevention and Research Institute of Texas (RR220038). C. Chad Quarles is a CPRIT Scholar in Cancer Research.].

Please state what role the funders took in the study.  If the funders had no role, please state: ""The funders had no role in study design, data collection and analysis, decision to publish, or preparation of the manuscript.""

If this statement is not correct you must amend it as needed.

Please include this amended Role of Funder statement in your cover letter; we will change the online submission form on your behalf.

3. We note that you have indicated that there are restrictions to data sharing for this study. For studies involving human research participant data or other sensitive data, we encourage authors to share de-identified or anonymized data. However, when data cannot be publicly shared for ethical reasons, we allow authors to make their data sets available upon request. For information on unacceptable data access restrictions, please see http://journals.plos.org/plosone/s/data-availability#loc-unacceptable-data-access-restrictions.

Before we proceed with your manuscript, please address the following prompts:

a) If there are ethical or legal restrictions on sharing a de-identified data set, please explain them in detail (e.g., data contain potentially identifying or sensitive patient information, data are owned by a third-party organization, etc.) and who has imposed them (e.g., a Research Ethics Committee or Institutional Review Board, etc.). Please also provide contact information for a data access committee, ethics committee, or other institutional body to which data requests may be sent.

b) If there are no restrictions, please upload the minimal anonymized data set necessary to replicate your study findings to a stable, public repository and provide us with the relevant URLs, DOIs, or accession numbers. Please see http://www.bmj.com/content/340/bmj.c181.long for guidelines on how to de-identify and prepare clinical data for publication. For a list of recommended repositories, please see https://journals.plos.org/plosone/s/recommended-repositories. You also have the option of uploading the data as Supporting Information files, but we would recommend depositing data directly to a data repository if possible.

Please update your Data Availability statement in the submission form accordingly.

4. Your ethics statement should only appear in the Methods section of your manuscript. If your ethics statement is written in any section besides the Methods, please move it to the Methods section and delete it from any other section. Please ensure that your ethics statement is included in your manuscript, as the ethics statement entered into the online submission form will not be published alongside your manuscript.

5. If the reviewer comments include a recommendation to cite specific previously published works, please review and evaluate these publications to determine whether they are relevant and should be cited. There is no requirement to cite these works unless the editor has indicated otherwise.

[Note: HTML markup is below. Please do not edit.]

Reviewers' comments:

Reviewer's Responses to Questions

**Comments to the Author**

1. Is the manuscript technically sound, and do the data support the conclusions?

Reviewer #1: Partly

Reviewer #2: Yes

**********

2. Has the statistical analysis been performed appropriately and rigorously?

Reviewer #1: Yes

Reviewer #2: Yes

**********

3. Have the authors made all data underlying the findings in their manuscript fully available?

The PLOS Data policy

Reviewer #1: No

Reviewer #2: No

**********

4. Is the manuscript presented in an intelligible fashion and written in standard English?

Reviewer #1: Yes

Reviewer #2: Yes

**********

Reviewer #1: This study integrates diffusion MRI–based brain age prediction, the DTI-ALPS index, white matter tract microstructure, and symptom burden to investigate brain aging characteristics and their potential influencing factors in long-term survivors of head and neck cancer. The research question has clinical relevance, the normative healthy cohort is relatively large, and the authors employed nested cross-validation, bias correction, and moderation analyses. The overall study design is somewhat innovative. However, the manuscript still has the following issues:

1.The DTI-ALPS index should not be directly equated with glymphatic system function or operational efficiency. The manuscript repeatedly uses terms such as “glymphatic function,” “glymphatic efficiency,” and “glymphatic clearance” to describe the meaning of ALPS, and states that DTI-ALPS can noninvasively quantify glymphatic system efficiency in vivo. In fact, DTI-ALPS is an indirect diffusion MRI metric calculated from directional diffusivities in specific periventricular white matter regions. The ALPS index may also be influenced by local white matter fiber architecture, brain atrophy, ventricular enlargement, white matter lesions, ROI placement, partial-volume effects, and diffusion acquisition parameters. The manuscript currently infers that the glymphatic system has a neuroprotective effect from associations of the ALPS index with BAG or fornix QA. This biological interpretation clearly exceeds what can be supported by this metric and by a cross-sectional study.

2.The patient cohort is defined by the authors as an external validation cohort. However, the final model was selected after comparing model performance in this cohort. Therefore, the patient data were effectively involved in model selection and no longer constitute a fully independent external test dataset.

3.Key clinical confounders were not included in the models. ALPS, white matter diffusion metrics, BAG, and symptom burden may all be influenced by tumor site, stage and HPV status, radiation dose, irradiation field and brain radiation exposure, hypertension, diabetes, dyslipidemia, and cardiovascular or cerebrovascular disease. The manuscript adjusts only for age, sex, and the MRI–MDASI interval, which is insufficient to exclude these shared causes. In particular, the authors repeatedly interpret the findings in terms of inflammatory, sleep-related, and vascular mechanisms, although these factors were neither directly measured nor controlled.

4.A positive mean BAG alone is insufficient to demonstrate accelerated brain aging in the patient group. The authors mainly infer accelerated brain aging from the mean BAG of 3.59 years generated by the random forest model in the patient group. However, the manuscript should further provide a formal test of whether BAG differs from zero, together with the corresponding 95% confidence interval.

5.The Methods section lacks key information required to ensure reproducibility.

6.There is a substantial temporal mismatch between MRI and symptom assessment. The MDASI evaluates symptoms during the preceding 24 hours, whereas the mean interval between MRI and MDASI assessment was 1.76 ± 4.83 years. Including the interval as a covariate does not resolve the lack of temporal alignment between the two measurements.

Reviewer #2: In this manuscript Bajaj et al have tested machine learning algorithms to test if survivors of head and neck cancer have accelerated brain aging and whether glymphatic function influences brain aging. They report accelerated brain aging and disruption of limbic white matter pathways in HN cancer survivors while glymphatic system function may have a protective effect.

The study addresses an important gap in oncology research by investigating the impact of cancer therapy on brain aging. The manuscript is well written but could benefit from minor revisions. Below are some specific comments and suggestions:

1. The authors should describe the method used for age range harmonization

2. The authors should discuss the clinical implications of their observations and how it can help improve patient care

3. The authors should discuss how the present brain age prediction model compares with previously published models in terms of methodology and predictive performance

4. Did the authors evaluate whether treatment modality, treatment duration, or cancer stage were associated with brain age gap or white matter microstructural measures?

5. Does the apparent protective association of glymphatic function diminish with increasing symptom burden, or does reduced glymphatic function simply reflect the extent of underlying brain injury, thereby serving as a marker rather than a mediator of symptom severity? The authors should comment on this.

6. The authors should indicate whether the model, code, or feature extraction pipeline are publicly available

**********

what does this mean?). If published, this will include your full peer review and any attached files.

If you choose “no”, your identity will remain anonymous but your review may still be made public.

**Do you want your identity to be public for this peer review?**  For information about this choice, including consent withdrawal, please see our Privacy Policy

Reviewer #1: No

Reviewer #2: **Yes:**  Reema Banarjee

**********

[NOTE: If reviewer comments were submitted as an attachment file, they will be attached to this email and accessible via the submission site. Please log into your account, locate the manuscript record, and check for the action link "View Attachments". If this link does not appear, there are no attachment files.]

To ensure your figures meet our technical requirements, please review our figure guidelines: https://journals.plos.org/plosone/s/figures

You may also use PLOS’s free figure tool, NAAS, to help you prepare publication quality figures: https://journals.plos.org/plosone/s/figures#loc-tools-for-figure-preparation.

NAAS will assess whether your figures meet our technical requirements by comparing each figure against our figure specifications.

# Author comment: Author response to Decision Letter 1

**DOI:** 10.1371/journal.pone.0360924.r003

24 Aug 2026

Response to Reviewers Comments

Manuscript ID: PONE-D-26-25905

Original Title: Lower symptom burden strengthens the association between glymphatic function and brain aging in head and neck cancer survivors

Revised Title: Lower symptom burden strengthens the association between DTI-ALPS index and brain aging in head and neck cancer survivors

Reviewer #1: This study integrates diffusion MRI–based brain age prediction, the DTI-ALPS index, white matter tract microstructure, and symptom burden to investigate brain aging characteristics and their potential influencing factors in long-term survivors of head and neck cancer. The research question has clinical relevance, the normative healthy cohort is relatively large, and the authors employed nested cross-validation, bias correction, and moderation analyses. The overall study design is somewhat innovative. However, the manuscript still has the following issues.

Response: We sincerely thank the reviewer for the positive and thoughtful assessment of our study. We agree that few aspects of the manuscript require further clarification and improvement. In response to the reviewer’s comments, we have carefully revised the manuscript. Detailed responses to each of the reviewer’s concerns are provided below, with corresponding changes highlighted in the revised manuscript.

Reviewer Comment 1. The DTI-ALPS index should not be directly equated with glymphatic system function or operational efficiency. The manuscript repeatedly uses terms such as “glymphatic function,” “glymphatic efficiency,” and “glymphatic clearance” to describe the meaning of ALPS, and states that DTI-ALPS can noninvasively quantify glymphatic system efficiency in vivo. In fact, DTI-ALPS is an indirect diffusion MRI metric calculated from directional diffusivities in specific periventricular white matter regions. The ALPS index may also be influenced by local white matter fiber architecture, brain atrophy, ventricular enlargement, white matter lesions, ROI placement, partial-volume effects, and diffusion acquisition parameters. The manuscript currently infers that the glymphatic system has a neuroprotective effect from associations of the ALPS index with BAG or fornix QA. This biological interpretation clearly exceeds what can be supported by this metric and by a cross-sectional study.

Response: We thank the reviewer for this important clarification. We agree that the DTI-ALPS index is an indirect diffusion MRI–based measure and should not be interpreted as a direct measure of glymphatic system function, efficiency, or clearance. We have therefore substantially revised the manuscript to ensure that our terminology and biological interpretations do not overstate what can be inferred from the ALPS index. Specifically, throughout the manuscript (including title), we have replaced definitive terms such as “glymphatic function,” “glymphatic efficiency,” and “glymphatic clearance” with more appropriate terminology, including “DTI-ALPS index” or “ALPS-derived measure,” where appropriate. We have also removed statements suggesting that DTI-ALPS can directly quantify glymphatic system efficiency in vivo. In addition, we explicitly acknowledge that the ALPS index may be influenced by factors beyond glymphatic-related processes, including local white matter fiber architecture, brain atrophy and ventricular enlargement, white matter lesions, ROI placement, partial-volume effects, and diffusion acquisition parameters. Importantly, we have revised our interpretation of the associations between ALPS and brain age gap or fornix QA. We no longer describe these associations as evidence that the glymphatic system has a neuroprotective effect. Instead, we interpret them as associations between an ALPS-derived diffusion metric and neuroimaging/symptom-related measures. Given the cross-sectional nature of the study, we also acknowledge that causal or mechanistic conclusions regarding glymphatic function, neuroprotection, or brain aging cannot be established.

These revisions have been made throughout the Abstract, Introduction, Material and Methods, Results, Discussion, and Limitations and Conclusion to ensure consistency and to appropriately frame the DTI-ALPS index as an indirect and potentially nonspecific diffusion MRI measure rather than a direct measurement of glymphatic function.

Reviewer Comment 2. The patient cohort is defined by the authors as an external validation cohort. However, the final model was selected after comparing model performance in this cohort. Therefore, the patient data were effectively involved in model selection and no longer constitute a fully independent external test dataset.

Response: We thank the reviewer for this important clarification. We agree that consideration of patient-cohort performance during final model selection means that the cohort should not be considered a completely untouched independent test set. However, the patient cohort was not used to train the models or estimate model parameters. Rather, we evaluated the candidate models in the patient cohort to identify the model that demonstrated the best generalization and the least systematic bias when transferred from the healthy control population to the head and neck cancer population. Model selection was therefore based on the overall generalizability and bias of the candidate models (lines 273-274 and lines 277-284) rather than on statistical significance or the magnitude of the observed patient effect. We have clarified this distinction in the revised manuscript and now refer to the patient cohort as an external evaluation/validation cohort (line 273), rather than a fully independent test set. We have also acknowledged that using patient-cohort performance to select the final model introduces a degree of data dependence and therefore limits the interpretation of the patient cohort as an unbiased final test dataset. Importantly, the selected model was not retrained using patient data, preserving its application as an externally derived model to the cancer cohort (lines 277-284).

Reviewer Comment 3. Key clinical confounders were not included in the models. ALPS, white matter diffusion metrics, BAG, and symptom burden may all be influenced by tumor site, stage and HPV status, radiation dose, irradiation field and brain radiation exposure, hypertension, diabetes, dyslipidemia, and cardiovascular or cerebrovascular disease. The manuscript adjusts only for age, sex, and the MRI–MDASI interval, which is insufficient to exclude these shared causes. In particular, the authors repeatedly interpret the findings in terms of inflammatory, sleep-related, and vascular mechanisms, although these factors were neither directly measured nor controlled.

Response: We thank the reviewer for this important comment. We agree that tumor characteristics, treatment exposure, and vascular/metabolic comorbidities may influence the observed neuroimaging and symptom measures. Our models were intentionally parsimonious and included prespecified covariates (age, sex, and MRI–MDASI interval) that were consistently available and directly relevant to the primary analyses. We did not include all potential clinical variables because several were incompletely available across participants, while others were highly interrelated (e.g., tumor characteristics and treatment variables). Including numerous correlated or incompletely measured variables could substantially reduce the analyzable sample, introduce selection bias, and result in unstable or potentially over-adjusted models. Moreover, the objective of this study was to characterize associations rather than estimate independent causal effects of specific tumor or treatment factors.

More specifically, we believe factors such as tumor characteristics, cancer stage, and treatment modality function more plausibly as mediators than confounders in this design — treatment and tumor burden likely lie on the causal pathway between HN cancer and brain-age deviation, rather than being independent common causes, and adjusting for them risks overadjustment bias that would mask the very effect we aim to characterize. In addition, with n = 135, several treatment subgroups contain only 5–10 participants (see Supplementary section S1), and tumor site/stage strongly predict treatment selection, so entering these as covariates would introduce collinearity and unstable estimates rather than improve confound control. Finally, our study's aim was to characterize brain-age deviation across the real-world heterogeneity of HN cancer survivorship rather than to isolate a treatment-independent effect and adjusting for treatment would work against this goal and reduce generalizability.

We acknowledge that this approach does not eliminate residual confounding. We have therefore added this limitation to the revised manuscript (lines 464-471) and substantially tempered our interpretation of inflammatory, sleep-related, vascular, and other potential mechanisms, which were not directly measured in this study (lines 422-424).

Reviewer Comment 4. A positive mean BAG alone is insufficient to demonstrate accelerated brain aging in the patient group. The authors mainly infer accelerated brain aging from the mean BAG of 3.59 years generated by the random forest model in the patient group. However, the manuscript should further provide a formal test of whether BAG differs from zero, together with the corresponding 95% confidence interval.

Response: We thank the reviewer for this important point. We agree that a positive mean BAG alone does not establish accelerated brain aging at the group level. We have therefore performed a formal one-sample test of BAG against zero in the patient cohort and now report the corresponding 95% confidence interval. The mean BAG was 3.59 (SD = 8.56) years (95% CI: [2.11, 5.06]), one-sample t-test: t[131] = 4.82, p < .001), indicating that the mean BAG was significantly greater than zero. These results have been added to the revised Results (lines 278-280).

Reviewer Comment 5. The Methods section lacks key information required to ensure reproducibility.

Response: We thank the reviewer for highlighting this important issue. We agree that additional methodological details are necessary to ensure transparency and reproducibility. We have therefore carefully revised and expanded the Methods section to provide greater detail regarding participant selection (lines 126-128, 130-131), MRI preprocessing (lines 165-175), diffusion-derived measures (lines 176-188) and age harmonization for brain age prediction model (lines 217-223), and specific covariate selection (lines 464-471). We have also clarified the relevant quality-control procedures (lines 165-175), and analysis parameters (lines 179-185). These revisions are intended to provide sufficient methodological detail to facilitate independent replication of the study.

Reviewer Comment 6. There is a substantial temporal mismatch between MRI and symptom assessment. The MDASI evaluates symptoms during the preceding 24 hours, whereas the mean interval between MRI and MDASI assessment was 1.76 ± 4.83 years. Including the interval as a covariate does not resolve the lack of temporal alignment between the two measurements.

Response: We thank the reviewer for highlighting this important limitation. Although we included the MRI–MDASI interval as a covariate to account for this temporal difference, we acknowledge that this adjustment cannot fully establish temporal alignment or eliminate the possibility that brain measures and symptom burden changed during the intervening period. We have therefore revised the manuscript to explicitly acknowledge the temporal mismatch as a limitation and have tempered our interpretation of the associations between neuroimaging measures and symptom burden (lines 464-471). We now emphasize that these findings should be interpreted as associations rather than evidence of contemporaneous or causal relationships. Future prospective studies with MRI and symptom assessments performed within a closely aligned time window will be important to confirm these findings.

Reviewer #2: In this manuscript Bajaj et al have tested machine learning algorithms to test if survivors of head and neck cancer have accelerated brain aging and whether glymphatic function influences brain aging. They report accelerated brain aging and disruption of limbic white matter pathways in HN cancer survivors while glymphatic system function may have a protective effect. The study addresses an important gap in oncology research by investigating the impact of cancer therapy on brain aging. The manuscript is well written but could benefit from minor revisions. Below are some specific comments and suggestions.

Response: We thank the reviewer for the positive and constructive assessment of our manuscript. In response to the reviewer’s suggestions, we have carefully revised the manuscript. We address each of the specific comments below.

Reviewer Comment 1. The authors should describe the method used for age range harmonization.

Response: We thank the reviewer for this suggestion. To harmonize the age ranges between the healthy control and HN cancer cohort, we used an age-range restriction approach rather than resampling or individual age matching. First, HN cancer participants outside the minimum-to-maximum age range of the healthy control cohort were excluded, reducing the cancer cohort from 135 to 132 participants. We then restricted the healthy control cohort to participants whose ages were within the resulting age range of the HN cohort, reducing the healthy control cohort from 1,228 to 1,176 participants. Thus, both cohorts were analyzed within a common overlapping age range, minimizing extrapolation of the brain age model beyond the age distribution represented in the study cohorts. This procedure has been clarified in the revised Methods section (lines 217-223).

Reviewer Comment 2. The authors should discuss the clinical implications of their observations and how it can help improve patient care.

Response: We thank the reviewer for this important comment. We have expanded the Discussion (lines 454-462) to clarify how our findings may contribute to patient care. The observed accelerated brain aging could potentially help identify HN cancer survivors with greater-than-expected alterations in brain health who may benefit from closer symptom monitoring. Incorporating brain age gap (BAG) with white matter measures and patient-reported symptom burden may further support individualized survivorship assessment and identification of patients who may benefit from targeted supportive or rehabilitative interventions. The association between higher DTI-ALPS index and lower BAG also provides a potential imaging marker for monitoring brain health. Although these applications require prospective validation, this framework could ultimately facilitate earlier identification of vulnerable survivors and more personalized survivorship care.

Reviewer Comment 3. The authors should discuss how the present brain age prediction model compares with previously published models in terms of methodology and predictive performance.

Response: We thank the reviewer for this important comment. We have expanded the Discussion to contextualize the methodology and predictive performance of our brain age model relative to previously published machine learning approaches. In particular, we now compare our most generalizable model (i.e., random forest (RF) model) with a systematic evaluation of 27 machine learning models across the Human Connectome Project (HCP), Cambridge Centre for Ageing and Neuroscience (Cam-CAN), and Information eXtraction from Images (IXI) datasets. While that prior work focused primarily on structural morphological measures, our model was developed using diffusion-derived measures. Brain age prediction using diffusion imaging features has been comparatively rarely investigated, despite diffusion measures providing complementary information about age-related alterations in white matter and tissue microstructure.

Our RF model demonstrated good generalization to an independent, unseen HCP test sample, with R=0.80, R2=0.64, MAE=7.32 years, and RMSE=9.12 years. Although the MAE was higher than that reported for models evaluated in the young-adult HCP sample in the prior study, it falls within the performance range reported for the broader-age Cam-CAN and IXI datasets. Direct comparisons should nevertheless be interpreted cautiously because of differences in imaging modality, age distributions, feature sets, and cross-validation/methodology procedures.

We further evaluated the model in an independent cancer cohort and observed evidence of accelerated brain aging in patients with HN cancer. The direction and magnitude of the observed accelerated-aging phenotype were broadly consistent with previous brain-age studies in cancer populations, despite the fact that much of the existing literature has focused on other cancer types rather than just head and neck cancer. Thus, the convergence of good predictive performance in an independent healthy test sample and biologically plausible accelerated-aging estimates in an external cancer cohort provides additional support for the utility of diffusion-based brain age modeling. These comparisons and considerations have now been incorporated into the revised Discussion (lines 387–413).

Reviewer Comment 4. Did the authors evaluate whether treatment modality, treatment duration, or cancer stage were associated with brain age gap or white matter microstructural measures?

Response: We thank the reviewer for this important question. Treatment modality, treatment duration, and cancer stage were not included as primary covariates in the brain age and white matter analyses because the study was designed primarily to characterize overall neuroimaging alterations in long-term head and neck cancer survivors rather than to evaluate treatment-specific effects. In addition, treatment-related variables were not uniformly available or sufficiently detailed across the cohort, and several treatment characteristics were highly interrelated with significant overlaps (see Supplementary section S1). We acknowledge that these factors may contribute to variability in BAG and white matter microstructure and have therefore added this issue as a limitation (lines 464-471). We have also tempered our interpretation to avoid attributing the observed neuroimaging alterations specifically to cancer treatment or disease stage. Future studies with comprehensive treatment and disease-characterization data will be needed to determine treatment- and stage-specific effects.

Reviewer Comment 5. Does the apparent protective association of glymphatic function diminish with increasing symptom burden, or does reduced glymphatic function simply reflect the extent of underlying brain injury, thereby serving as a marker rather than a mediator of symptom severity? The authors should comment on this.

Response: We thank the reviewer for this insightful comment. We agree that the observed associations cannot distinguish whether the DTI-ALPS index represents a protective factor, a marker of underlying brain injury, or a correlate of symptom burden. Because our study is cross-sectional and DTI-ALPS is an indirect diffusion MRI measure, we cannot establish mediation or directionality. Our moderation analyses should not be interpreted as evidence of protective association. Reduced ALPS may alternatively reflect broader white matter or brain alterations associated with greater symptom burden. We have therefore revised our manuscript thoroughly to explicitly acknowledge these alternative interpretations and now describe the ALPS-related findings as moderating associations rather than evidence of a protective or mediating glymphatic effect. Longitudinal studies with repeated imaging and symptom assessments will be necessary to determine the temporal and causal relationships among ALPS, brain injury, and symptom burden.

Reviewer Comment 6. The authors should indicate whether the model, code, or feature extraction pipeline are publicly available.

Response: We thank the reviewer for this suggestion. The study-specific code, model, and feature-extraction pipeline are not currently publicly available because the analyses were developed and executed within our institution using clinical research data subject to institutional data-use, privacy, and governance requirements. In particular, the underlying clinical and neuroimaging data cannot be publicly distributed, and the associated analysis pipeline contains dataset-specific processing and feature-extraction procedures. We have clarified this in the revised Data Availability statement that de-identified data, appropriate analysis code and documentation may be made available from the University of Texas MD Anderson Cancer Center and the corresponding author only upon reasonable request, subject to institutional policies and applicable data-use restrictions.

Attachment Submitted filename: Response to Reviewers.docx

# Peer review: Decision Letter 1

**DOI:** 10.1371/journal.pone.0360924.r004

**Authors:** Feng Liu

17 Sep 2026

Dear Dr. Bajaj,

Thank you for submitting your manuscript to PLOS One. After careful consideration, we feel that it has merit but does not fully meet PLOS One’s publication criteria as it currently stands. Therefore, we invite you to submit a revised version of the manuscript that addresses the points raised during the review process.

Please submit your revised manuscript by Oct 17 2026 11:59PM. If you will need more time than this to complete your revisions, please reply to this message or contact the journal office at plosone@plos.org. When you're ready to submit your revision, log on to https://www.editorialmanager.com/pone/ and select the 'Submissions Needing Revision' folder to locate your manuscript file.

- A letter that responds to each point raised by the academic editor and reviewer(s). You should upload this letter as a separate file labeled 'Response to Reviewers'.
- A marked-up copy of your manuscript that highlights changes made to the original version. You should upload this as a separate file labeled 'Revised Manuscript with Track Changes'.
- An unmarked version of your revised paper without tracked changes. You should upload this as a separate file labeled 'Manuscript'.

If you would like to make changes to your financial disclosure, please include your updated statement in your cover letter. Guidelines for resubmitting your figure files are available below the reviewer comments at the end of this letter.

If applicable, we recommend that you deposit your laboratory protocols in protocols.io to enhance the reproducibility of your results. Protocols.io assigns your protocol its own identifier (DOI) so that it can be cited independently in the future. For instructions see: https://journals.plos.org/plosone/s/submission-guidelines#loc-laboratory-protocols. Additionally, PLOS One offers an option for publishing peer-reviewed Lab Protocol articles, which describe protocols hosted on protocols.io. Read more information on sharing protocols at https://plos.org/protocols?utm_medium=editorial-email&utm_source=authorletters&utm_campaign=protocols.

As the corresponding author, your ORCID iD is verified in the submission system and will appear in the published article. PLOS supports the use of ORCID, and we encourage all coauthors to register for an ORCID iD and use it as well. Please encourage your coauthors to verify their ORCID iD within the submission system before final acceptance, as unverified ORCID iDs will not appear in the published article. *Only*  the individual author can complete the verification step; PLOS staff *cannot*  verify ORCID iDs on behalf of authors.

We look forward to receiving your revised manuscript.

Kind regards,

Feng Liu, Ph.D.

Academic Editor

PLOS One

Journal Requirements:

If the reviewer comments include a recommendation to cite specific previously published works, please review and evaluate these publications to determine whether they are relevant and should be cited. There is no requirement to cite these works unless the editor has indicated otherwise.

Please review your reference list to ensure that it is complete and correct. If you have cited papers that have been retracted, please include the rationale for doing so in the manuscript text, or remove these references and replace them with relevant current references. Any changes to the reference list should be mentioned in the rebuttal letter that accompanies your revised manuscript. If you need to cite a retracted article, indicate the article’s retracted status in the References list and also include a citation and full reference for the retraction notice.

[Note: HTML markup is below. Please do not edit.]

Reviewers' comments:

Reviewer's Responses to Questions

**Comments to the Author**

Reviewer #1: (No Response)

Reviewer #2: All comments have been addressed

**********

2. Is the manuscript technically sound, and do the data support the conclusions?

Reviewer #1: (No Response)

Reviewer #2: Yes

**********

3. Has the statistical analysis been performed appropriately and rigorously?

Reviewer #1: (No Response)

Reviewer #2: Yes

**********

4. Have the authors made all data underlying the findings in their manuscript fully available?

The PLOS Data policy

Reviewer #1: (No Response)

Reviewer #2: Yes

**********

5. Is the manuscript presented in an intelligible fashion and written in standard English?

Reviewer #1: (No Response)

Reviewer #2: Yes

**********

Reviewer #1: The authors have made substantial efforts to address the comments raised in the previous round. In particular, the terminology surrounding the DTI-ALPS index has been revised, additional methodological details have been provided, and a formal statistical test of whether the mean brain age gap (BAG) differed from zero has been added. These revisions have improved the manuscript. However, the following issue remains unresolved:

Although the authors now acknowledge that the patient cohort contributed to final model selection and have redefined it as an external evaluation/validation cohort, this revision does not resolve the data dependence introduced by the model-selection procedure. Of particular concern, ENET showed better predictive performance than RF in the independent held-out HC test set (R² = 0.76, MAE = 5.80 years vs. R² = 0.64, MAE = 7.32 years), whereas the two models produced BAG estimates in opposite directions when applied to the same HN cancer cohort: the mean BAG was −1.64 years for ENET but +3.59 years for RF. The authors subsequently selected RF on the basis of its generalizability and systematic bias in the patient cohort and then used the positive BAG generated by RF to support the central conclusion that “HN survivors exhibit accelerated brain aging”; the revised manuscript also explicitly acknowledges that patient-cohort performance was considered during final model selection. Therefore, the issue is no longer merely whether this cohort can be considered an independent external test set; rather, the central conclusion of accelerated brain aging appears to be materially dependent on the choice of prediction model. The authors’ statement that RF was not retrained using patient data does not eliminate this post-selection bias. The final model should ideally be selected using criteria completely independent of the HN cohort, for example, based only on the HC training data, nested cross-validation, and the held-out HC test set, and then applied once to the HN cohort after model selection has been finalized. Alternatively, the authors should report the ENET and RF findings in parallel and explicitly acknowledge that the conclusion of accelerated brain aging is not robust to model choice. Under the current analytical framework, the positive BAG obtained from an RF model selected after evaluating its performance in the patient cohort does not provide sufficiently independent evidence to support the conclusion of accelerated brain aging.

Reviewer #2: In response to the reviewers' comments, the authors have made significant changes in the manuscript and added new discussion that adequately outlines the limitations and possible future clinical applications of the study. The manuscript is now acceptable for publication.

**********

what does this mean?). If published, this will include your full peer review and any attached files.

If you choose “no”, your identity will remain anonymous but your review may still be made public.

**Do you want your identity to be public for this peer review?**  For information about this choice, including consent withdrawal, please see our Privacy Policy

Reviewer #1: No

Reviewer #2: **Yes:**  Reema Banarjee

**********

[NOTE: If reviewer comments were submitted as an attachment file, they will be attached to this email and accessible via the submission site. Please log into your account, locate the manuscript record, and check for the action link "View Attachments". If this link does not appear, there are no attachment files.]

To ensure your figures meet our technical requirements, please review our figure guidelines: https://journals.plos.org/plosone/s/figures

You may also use PLOS’s free figure tool, NAAS, to help you prepare publication quality figures: https://journals.plos.org/plosone/s/figures#loc-tools-for-figure-preparation.

NAAS will assess whether your figures meet our technical requirements by comparing each figure against our figure specifications.

# Author comment: Author response to Decision Letter 2

**DOI:** 10.1371/journal.pone.0360924.r005

21 Sep 2026

Response to Reviewers Comments

Manuscript ID: PONE-D-26-25905

Title: Lower symptom burden strengthens the association between DTI-ALPS index and brain aging in head and neck cancer survivors

Reviewer #1

Comment 1A: The authors have made substantial efforts to address the comments raised in the previous round. In particular, the terminology surrounding the DTI-ALPS index has been revised, additional methodological details have been provided, and a formal statistical test of whether the mean brain age gap (BAG) differed from zero has been added. These revisions have improved the manuscript.

Response: We sincerely thank the reviewer for the positive assessment of our revised version.

Comment 1B: However, the following issue remains unresolved. Although the authors now acknowledge that the patient cohort contributed to final model selection and have redefined it as an external evaluation/validation cohort, this revision does not resolve the data dependence introduced by the model-selection procedure. Of particular concern, ENET showed better predictive performance than RF in the independent held-out HC test set (R² = 0.76, MAE = 5.80 years vs. R² = 0.64, MAE = 7.32 years), whereas the two models produced BAG estimates in opposite directions when applied to the same HN cancer cohort: the mean BAG was −1.64 years for ENET but +3.59 years for RF. The authors subsequently selected RF on the basis of its generalizability and systematic bias in the patient cohort and then used the positive BAG generated by RF to support the central conclusion that “HN survivors exhibit accelerated brain aging”; the revised manuscript also explicitly acknowledges that patient-cohort performance was considered during final model selection. Therefore, the issue is no longer merely whether this cohort can be considered an independent external test set; rather, the central conclusion of accelerated brain aging appears to be materially dependent on the choice of prediction model. The authors’ statement that RF was not retrained using patient data does not eliminate this post-selection bias. The final model should ideally be selected using criteria completely independent of the HN cohort, for example, based only on the HC training data, nested cross-validation, and the held-out HC test set, and then applied once to the HN cohort after model selection has been finalized. Alternatively, the authors should report the ENET and RF findings in parallel and explicitly acknowledge that the conclusion of accelerated brain aging is not robust to model choice. Under the current analytical framework, the positive BAG obtained from an RF model selected after evaluating its performance in the patient cohort does not provide sufficiently independent evidence to support the conclusion of accelerated brain aging.

Response: We thank the reviewer for raising this important concern regarding model selection and post-selection dependence. We greatly appreciate the reviewer’s careful consideration of this issue, which prompted us to re-examine our analytical framework and substantially clarify how the two brain age models are interpreted. We agree with the fact that the RF model was originally considered for subsequent analyses based partly on its performance in the HN cohort introduces data dependence and that the fact that the model was not retrained using patient data does not, by itself, eliminate this concern. Following the reviewer’s recommendation, we have therefore revised the analysis and manuscript to report the ENET and RF models in parallel rather than treating the HN cohort as an independent dataset for selecting a single final model. We now present the predictive performance and BAG estimates from both models, as well as their respective associations with ALPS and symptom burden.

As reported before, ENET showed superior predictive performance in the held-out HC test set, whereas RF showed stronger generalization in the external HN cohort compared with. The models also yielded different BAG estimates in the HN cohort: ENET produced a negative, nonsignificant BAG, whereas RF produced a positive, significant BAG. In the revised manuscript. we further examined whether these model-dependent BAG estimates showed convergent biological relationships with the ALPS index. Notably, the ENET-derived BAG did not demonstrate a significant association with ALPS in the omnibus regression models for any of the four symptom-burden domains after correction for multiple comparisons, nor were the ALPS × symptom-burden interactions significant in the corresponding moderation analyses. In contrast, the RF-derived BAG demonstrated significant ALPS associations in the omnibus regression analyses across all four symptom-burden domains after multiple-comparison correction, with significant ALPS × symptom-burden interactions observed for two of the four domains after correction. Feature-level analyses further demonstrated biologically coherent ALPS–WM microstructure relationships involving WM pathways identified as key contributors to the RF brain age model.

Based on these parallel analyses, we have revised our interpretation to distinguish predictive model performance from biological relevance in the target clinical population. We do not claim that RF is universally superior to ENET, nor do we use the HN cohort to retrospectively establish RF as the sole optimal prediction model. Rather, the two models were carried forward because they provide complementary information: ENET, a predominantly linear model, showed stronger internal predictive performance in held-out healthy controls, whereas RF, which can capture nonlinear and higher-order relationships, demonstrated stronger external generalizability and greater biological relevance in the HN cohort. The consistency of the RF-derived BAG with the ALPS and symptom-related findings provided the basis for interpreting the positive RF BAG as evidence of accelerated brain aging in the HN cohort.

We have also explicitly tempered the conclusion in the revised manuscript. Specifically, we now acknowledge that the direction and magnitude of BAG were not fully consistent across the two modeling approaches and that the evidence for accelerated brain aging is therefore model-dependent rather than uniformly robust across models. The conclusion of accelerated brain aging is based on the model that demonstrated stronger generalization to the clinical population and greater biological relevance within the HN cohort, while the ENET findings are reported transparently rather than being omitted or treated as a failed model. We believe this revised approach more appropriately reflects the uncertainty associated with applying brain age models developed in healthy populations to clinical cohorts and avoids presenting the RF-derived BAG as an independently validated finding.

In the revised manuscript, we now report both Bonferroni corrected ENET and RF results throughout the relevant Results and Discussion sections and explicitly discusses the implications of model choice, external generalizability, and the differing functional forms of the two algorithms. All new results, corresponding text, and related edits throughout the manuscript have been highlighted in the revised version. The relevant tables have also been revised to include the ENET results, and new figures have been added to present the ENET brain age model results and associated findings. These changes provide a transparent parallel presentation of both modeling approaches and directly address the reviewer’s concern regarding model-selection dependence.

Attachment Submitted filename: Response_Reviewers_Updated.docx

# Peer review: Decision Letter 2

**DOI:** 10.1371/journal.pone.0360924.r006

**Authors:** Feng Liu

5 Oct 2026

Lower symptom burden strengthens the association between DTI-ALPS index and brain aging in head and neck cancer survivors

PONE-D-26-25905R2

Dear Dr. Bajaj,

We’re pleased to inform you that your manuscript has been judged scientifically suitable for publication and will be formally accepted for publication once it meets all outstanding technical requirements.

Within one week, you’ll receive an e-mail detailing the required amendments. When these have been addressed, you’ll receive a formal acceptance letter and your manuscript will be scheduled for publication.

An invoice will be generated when your article is formally accepted. Please note, if your institution has a publishing partnership with PLOS and your article meets the relevant criteria, all or part of your publication costs will be covered. Please make sure your user information is up-to-date by logging into Editorial Manager at Editorial Manager® and clicking the ‘Update My Information' link at the top of the page. For questions related to billing, please contact billing support.

If your institution or institutions have a press office, please notify them about your upcoming paper to help maximize its impact. If they’ll be preparing press materials, please inform our press team as soon as possible -- no later than 48 hours after receiving the formal acceptance. Your manuscript will remain under strict press embargo until 2 pm Eastern Time on the date of publication. For more information, please contact onepress@plos.org.

Kind regards,

Feng Liu, Ph.D.

Academic Editor

PLOS One

Additional Editor Comments (optional):

Reviewers' comments:

Reviewer's Responses to Questions

**Comments to the Author**

Reviewer #1: (No Response)

**********

2. Is the manuscript technically sound, and do the data support the conclusions?

Reviewer #1: (No Response)

**********

3. Has the statistical analysis been performed appropriately and rigorously?

Reviewer #1: (No Response)

**********

4. Have the authors made all data underlying the findings in their manuscript fully available?

The PLOS Data policy

Reviewer #1: (No Response)

**********

5. Is the manuscript presented in an intelligible fashion and written in standard English?

Reviewer #1: (No Response)

**********

Reviewer #1: (No Response)

**********

what does this mean?). If published, this will include your full peer review and any attached files.

If you choose “no”, your identity will remain anonymous but your review may still be made public.

**Do you want your identity to be public for this peer review?**  For information about this choice, including consent withdrawal, please see our Privacy Policy

Reviewer #1: No

**********

# Editor report: Acceptance letter

**DOI:** 10.1371/journal.pone.0360924.r007

**Authors:** Feng Liu

PONE-D-26-25905R2

PLOS One

Dear Dr. Bajaj,

I'm pleased to inform you that your manuscript has been deemed suitable for publication in PLOS One. Congratulations! Your manuscript is now being handed over to our production team.

At this stage, our production department will prepare your paper for publication. This includes ensuring the following:

* All references, tables, and figures are properly cited

* All relevant supporting information is included in the manuscript submission,

* There are no issues that prevent the paper from being properly typeset

You will receive further instructions from the production team, including instructions on how to review your proof when it is ready. Please keep in mind that we are working through a large volume of accepted articles, so please give us a few days to review your paper and let you know the next and final steps.

Lastly, if your institution or institutions have a press office, please let them know about your upcoming paper now to help maximize its impact. If they'll be preparing press materials, please inform our press team within the next 48 hours. Your manuscript will remain under strict press embargo until 2 pm Eastern Time on the date of publication. For more information, please contact onepress@plos.org.

You will receive an invoice from PLOS for your publication fee after your manuscript has reached the completed accept phase. If you receive an email requesting payment before acceptance or for any other service, this may be a phishing scheme. Learn how to identify phishing emails and protect your accounts at https://explore.plos.org/phishing.

If we can help with anything else, please email us at customercare@plos.org.

Thank you for submitting your work to PLOS One and supporting open access.

Kind regards,

PLOS One Editorial Office Staff

on behalf of

Dr. Feng Liu

Academic Editor

PLOS One
